# Using the Newcomb–Benford law to study the association between a country’s COVID-19 reporting accuracy and its development

**DOI:** 10.1038/s41598-021-02367-z

**Published:** 2021-11-25

**Authors:** Vadim S. Balashov, Yuxing Yan, Xiaodi Zhu

**Affiliations:** 1 Rutgers School of Business-Camden, Camden, NJ 08102 USA; 2 SUNY at Geneseo, Geneseo, NY 14454 USA; 3 New Jersey City University, Jersey City, NJ 07305 USA

**Keywords:** Statistical methods, Infectious diseases, Epidemiology

## Abstract

The COVID-19 pandemic has spurred controversies related to whether countries manipulate reported data for political gains. We study the association between accuracy of reported COVID-19 data and developmental indicators. We use the Newcomb–Benford law (NBL) to gauge data accuracy. We run an OLS regression of an index constructed from developmental indicators (democracy level, gross domestic product per capita, healthcare expenditures, and universal healthcare coverage) on goodness-of-fit measures to the NBL. We find that countries with higher values of the developmental index are less likely to deviate from the Newcomb-Benford law. The relationship holds for the cumulative number of reported deaths and total cases but is more pronounced for the death toll. The findings are robust for second-digit tests and for a sub-sample of countries with regional data. The NBL provides a first screening for potential data manipulation during pandemics. Our study indicates that data from autocratic regimes and less developed countries should be treated with more caution. The paper further highlights the importance of independent surveillance data verification projects.

## Introduction

On March 11, 2020, the World Health Organization (WHO) declared the novel coronavirus disease 2019 (COVID-19) a pandemic. With tens of millions of confirmed cases and millions of deaths, this pandemic has spurred a great number of controversies, including many related to the accuracy of the data countries report. Mass media organizations around the globe argue that many countries have continued to manipulate the data for political or other gains^1–8^.

In this paper, we study the association between the accuracy of COVID-19 data reported by countries and their macroeconomic and political indicators. Our results show that countries that are more functional democracies, have higher income, and stronger healthcare systems report more accurate data. The relationship exists for the cumulative number of confirmed cases and for the cumulative number of reported deaths; however, the results are more pronounced for the number of deaths.

To gauge data accuracy, we use compliance with the Newcomb–Benford law (NBL), which is an observation that in many naturally occurring collections of numbers the first digit is not uniformly distributed. The numeral “1” will be the leading digit around one-third of the time; the numeral “2” will be the leading digit 18% of the time; and each subsequent numeral, “3” through “9,” will be the leading digit with decreasing frequency. One property of the NBL is that manipulated or fraudulent data deviate significantly from the theoretical NBL distribution. Due to the ease of its application and straightforward approach, the law has been extensively used to detect fraud and data manipulations. It has been applied to accounting, finance, macroeconomic, and forensic data to test for data manipulation and fraud. We apply the NBL to COVID-19 data for 185 countries affected by the pandemic. For each country, we first identify the period of exponential growth when the data are expected to obey the NBL. After the country’s data reach a plateau, the number of cases stabilizes, and the data are not expected to obey the NBL. During the growth period for each country, we calculate goodness-of-fit measures to estimate compliance with the NBL and use these measures as proxies for data accuracy.

We then study the relationship between our proxies for accuracy of data and indicators of the strength of the economy, democratic institutions, and healthcare systems. Specifically, we use the regression analysis to find the association between goodness-of-fit measures and an index constructed from four developmental indicators: the *Economist Intelligence Unit* Democracy Index, the gross domestic product per capita, healthcare expenditures as percentage of GDP, and the Universal Health Coverage Index.

Our main hypothesis is that countries with weaker democracies, and weaker economic and healthcare systems will have lower data accuracy as measured by the NBL goodness-of-fit statistic. Our results in many tests support the hypothesis. We find that our goodness-of-fit measures (which measure deviations from the theoretical distribution as given by the NBL) are negatively correlated with the developmental index and each of the macroeconomic indicators (Democracy Index, GDP, healthcare expenditures, and UHC). The results are true for the cumulative number of cases and the cumulative number of reported deaths. We find no results for the number of cured cases or conducted tests. We also find the result is more pronounced for the reported number of deaths than for the number of confirmed cases. We also find that deviations from the NBL are higher for the death toll. This indicates that, on average, autocratic regimes and poorer countries are more prone to misreport death tolls than the total number of citizens infected.

We conduct a series of robustness tests and find that our results are not driven by the specific period in which we calculate the goodness-of-fit measures, by small countries, by countries with a small number of cases or deaths, or by countries with extreme deviations from the NBL. We also show that the same relationship between proxies for accuracy of data and the developmental index is observed when we apply the NBL to second digits. One concern of our study is that the proxies for data accuracy are calculated based on limited sample sizes for individual countries. To resolve this potential problem, we confirm our findings for the sub-sample of 50 countries that provide regional data (at a state or province level). Regional data increase the sample size from which we calculate our statistics substantially and heighten the precision of our accuracy measures.

There is substantial body of literature assessing the tendency of misreporting COVID-19 surveillance pandemic data by countries using different statistical techniques, such as case fatality rates, excess mortality rates, the variance of reported data, the clustering of data, and even trends in search engines^9–13^. The inherent problem with these methods is their reliance on uniform approaches to measuring confirmed cases and COVID-19-related deaths across countries. Even though countries are expected to follow the same guidelines provided by the WHO when reporting cases, many variations exist (and sometimes appear in states and regions within a country) in how they collect and report data. Any comparisons of raw numbers-like the total number of confirmed cases, the number of deaths, and mortality rates-among countries may be driven by the difference in the number of tests conducted, the strength of the healthcare system, demographic composition, and reporting standards. Correct comparisons would require controlling for all those hard-to-observe variables. One helpful property of the NBL is its tolerance to different data generating processes between countries and, in contrast, its sensitivity to human intervention and manipulation of data in otherwise naturally occurring processes. This means that we can apply the test even if countries differ in how they measure COVID-19 cases and related deaths. The test is also free of country-specific differences, including public policies used to stop the pandemic, like quarantines, social distancing, testing, and availability of treatment.

There are some caveats with using the NBL. The NBL is an empirical observation. Departures from something only empirically observed should be treated with caution and do not by default mean causation. The test does not provide a conclusive evidence of intentional data falsification by a country’s government. In applications, the NBL is usually used as a first filter for detecting fraud. Deviations from the NBL simply mean that further investigation is needed to identify causes of such anomalies, which can be deliberate or not. Our results simply indicate that data from autocracies and poorer countries should be trusted less and are of lower accuracy. It should also be noted that the NBL test is not directional. However, it is unreasonable to believe that the government would willingly manipulate data to inflate the number of cases or deaths. Neither does the divergence from the NBL provide us specifics on how the data are being manipulated. We cannot ex ante predict which first digits will be over- or under-inflated. For example, if the country’s true number of cases is in the 2000s and the government tries to falsify data to look smaller and reports high 1900s, the first digit “one” will be over-represented in this country’s statistics. However, if the country’s numbers are in the 1000s and the governments falsifies data into the 900s, then the first digit “one” will be under-represented.

We stress that our paper is also not aimed to answer whether particular country’s data do not conform with the NBL. We indicate that such tests are problematic because they largely depend on the sample size and selected cutoff values for significance. We calculate goodness-of-fit measures for all countries, we then compare countries cross-sectionally and study the association of deviations from the NBL and the developmental indices.

Our paper contributes to the literature in several ways. First, our paper uses the NBL to assess the accuracy of COVID-19 reported data. Second, our study shows which data, if any, countries are more likely to misreport. To the extent that misreporting data is associated with deliberate data manipulation, our data are consistent with governments tending to downplay the news with the highest negative impact, i.e., the death toll, to the highest degree. To a slightly lower degree, countries tend to manipulate the total number of confirmed cases. We find no indication, on average, of systematic data errors for the number of conducted COVID-19 tests and the number of cured cases. Finally, we are the first study to document the cross-sectional link between macroeconomic and political regime indicators and the tendency to misreport data during pandemics. We show that authoritarian regimes and countries with low GDP per capita are more likely to deviate from the NBL. Overall, this study provides additional evidence of the link between developmental indicators and data accuracy that is often taken for granted.

Our study has broad implications. First, we provide evidence that the data supplied during pandemics may be of low quality, especially from autocracies and poorer countries, and we suggest that caution should be used when interpreting and using the data. Second, the study highlights the importance of initiatives to externally verify data provided by governments, including independent surveillance data verification projects. An example of such a project for economic data would be the Billion Prices Project (BPP) by Alberto Cavallo and Roberto Rigobon at MIT Sloan and Harvard Business School. Finally, we provide new evidence on the applicability of the NBL to detect data errors during pandemics.

## Literature review and hypotheses development

Studies have long posed questions about whether more developed countries provide more reliable data to the public than less developed countries in both theoretical and empirical settings. Extant studies show in other settings that countries with weaker democracies are more likely to manipulate data^14–18^ and have lower transparency^19–27^. These studies find that it is authoritarian regimes that are more vulnerable to negative information and have more incentives to distort and manipulate information that undermines their image. In addition, such regimes usually have control over mass media organizations and therefore have more capabilities to exercise control. Autocrats use data manipulation to improve their public image and prolong their stay in office. Rozenas and Stukal^27^ propose that autocrats are more likely to manipulate data for which it is more difficult for citizens to obtain hard external information benchmarks. COVID-19 provides a unique setting to test a related hypothesis. Pandemic surveillance data are hard to acquire independently by citizens because they lack access to the necessary large-scale data collection and medical facilities. At the same time, the news that the disease is raging and is widespread under authoritarian rule would be an indicator of the inefficiency or failure of the government. The death toll is even more damaging to the image of the autocrat, who sees such news as a threat and tries to downplay the scale of the problem.

Not all errors in data are deliberate and result from fraud. Data accuracy has been shown to be linked to the strength of the economy of a country. Hollyer et al.^24^ maintain that GDP per capita is a measure of the “*ability* of the governments to collect and disseminate high-quality statistical data.” In a similar vein, Judge and Schechter^28^ analyze the quality of survey data using the NBL and find that survey data in developing countries is of poor quality while data from developed countries is of better quality. We therefore formulate the following two hypotheses:

*Hypothesis 1: More developed countries are less likely to misreport pandemic surveillance data.*

*Hypothesis 2: The link between developmental indicators and data accuracy is more pronounced for the reported death toll.*

To gauge data accuracy, we use compliance with the NBL. We are not the only paper to use the NBL to test the validity of reported data during COVID-19^29–33^. However, many papers usually select one or a few countries and apply the NBL to test if there is any evidence of manipulation in a given country’s data. The authors claim that significant deviations from the NBL indicate data manipulation. They then use the cutoff values from the chi-squared or similar distributions and give a “yes-or-no” type of answer to their binary research question. These test statistics and inference results greatly depend on the sample size and selected cutoff values^34^. With large enough sample sizes, the null hypothesis of compliance with the NBL will be rejected in almost every case. Some studies estimate their test statistic at the country level, some studies estimate it at a regional or state level, and some studies use county-level data. At the same time, different studies use different cutoff values when deeming a distribution as “not-conforming” to the NBL. This leads to contradictory findings among these studies even when looking at the same country. We avoid these “yes-or-no” tests. Instead, we employ the same approach for all countries to calculate the test statistic. We use this test statistic as comparative levels of conformance and study the link between deviations from the NBL and the developmental index. Our study does not make any conclusions based on individual test statistics and in regards to individual countries’ misreporting. Instead, we make any inferences from the NBL test only in comparison. To our best knowledge, this is the first paper to examine the cross-section of deviations from the NBL across countries and compare them based on the developmental index when analyzing data errors during pandemics.

Not many studies apply the NBL in an international setting, though there are several notable exceptions. Nye and Moul^35^ indicate that international macroeconomic data generally conforms the NBL. They find, however, that for non-OECD (African) countries, the data do not conform with the law, which raises questions about data quality and manipulation in these countries. Gonzalez-Garcia^36^ uses a similar approach to test the annual IMF data, but finds no connection between independent assessments of data quality and adherence to the first-digit NBL in different country groups. Michalski and Stoltz^37^ provide a theoretical model and empirical findings that some countries strategically manipulate their economic data for short-term government gains. We contribute to this body of literature by applying the NBL to the pandemic data in the international setting and providing additional evidence that some types of countries are more likely to misreport not only macroeconomic data but also surveillance data during pandemics.

## Newcomb–Benford law of anomalous numbers

In many naturally occurring processes, the resulting data have the leading significant digit that is not uniformly distributed. The distribution is monotonically decreasing, with “1” being the most common first digit, and “9” being the least common. The law was formally stated by Newcomb^38^ and Benford^39^. A set of numbers is said to follow the NBL if the first digit *d* occurs with probability $P(d)=log_{10}(1+\frac{1}{d}).$ The law can be extended to digits beyond the first. In general, for the $n\text {th}$ digit, $n\ge 2$, the probability is given by $P(d)=\mathop {\sum _{k=10^{n-2}}^{10^{n-1}-1}log_{10}(1+\frac{1}{10k+d}).}$ This gives the following probabilities for observing the first and second digits:

**Table:** 

Digit	0	1	2	3	4	5	6	7	8	9
First	–	30.1%	17.6%	12.5%	9.7%	7.9%	6.7%	5.8%	5.1%	4.6%
Second	12.0%	11.4%	10.9%	10.4%	10.0%	9.7%	9.3%	9.0%	8.8%	8.5%

The NBL accurately describes many real-life sets of numerical data, including lengths of rivers, stock prices, street addresses, accounting data, populations, physical constants, and regression coefficients^40^. Data generated from many distributions and integer sequences have been shown to closely obey the NBL, including Fibonacci numbers, powers of numbers, exponential growth, many ratio distributions, and the *F*-distribution with low degrees of freedom^41–45^.

Not all distributions generate data that follow the law. For example, uniform distribution, normal distribution, and square roots of numbers do not obey it. For the data to obey the NBL, several criteria should be satisfied^40,46,47^: (a) data span several orders of magnitude and are relatively uniform over such orders; (b) the mean is greater than the median, with a positive skewness; (c) naturally occurring processes, the data which are the result of multiplicative fluctuations, and data that is not influenced by human intervention.

The last requirement, i.e., the fact that human intervention usually generates data that violates the NBL, has led to its usefulness in detecting fraud and data manipulation. Studies have shown that when humans intervene with the data generating process that is expected to comply with the NBL, compliance stops. For example, Diekmann^40^ and Horton et al.^48^ show that when scientific data are fabricated, they do not conform with the NBL. Cantu and Saiegh^49^ and Breunig and Goerres^50^ reveal the same effect for electoral data. Kaiser^51^ uncovers how discrepancies from the target NBL distribution can be used to test reliability among survey data sets.

Overall, the NBL has been used to detect fraud in: (a) scientific studies^28, 40, 48, 52^, (b) accounting^46, 48, 53–56^, (c) macroeconomic data^35–37, 57–61^, (d) forensic analysis^62^, (e) tax evasion^63, 64^, (f) toxic release inventory^65^, (g) reported data during pandemics^29, 30, 66, 67^.

Another useful property of the data obeying the NBL is that it is scale invariant, i.e., it is independent of the measurement units. This makes it a powerful tool when testing data from different sources (i.e., countries, companies). The NBL is also not the same as the imprecision (or variance) of the data. The data may well be very noisy but still is expected to conform with the law. For example, if a country’s data are collected with error, the first digit should still adhere to the NBL, as long as the error term is random with zero mean. In our application, it means that countries may differ in the way they count COVID-19 cases or deaths, but as long as the data for each country is expected to obey the NBL, we can test the data for the goodness-of-fit to the NBL.

## Data and variables

### Goodness-of-fit measures

To measure how well the data comply with the NBL, we use several goodness-of-fit measures. The most intuitive and commonly used is the chi-squared statistic:

1 $$\begin{aligned} Chi-\text {sq.}=\mathop {\sum _{d=1}^{9}\frac{(O_{d}-E_{d})^{2}}{E_{d}}}, \end{aligned}$$

where $O_{d}$ and $E_{d}$ are observed and expected by the NBL frequencies for digit *d*, respectively. Chi-squared, however, has several problems: it has low statistical power when used with small sample sizes and enormous power with large sample sizes. An alternative measure of goodness-of-fit proposed in extant studies is a modified version of the Kuiper^68^ test proposed by Stephens^69^ and Giles^70^ that is less dependent on the sample size *N*:

2 $$\begin{aligned} Kuiper= & {} (D^{+}+D^{-})\left[ \sqrt{N}+0.155+\frac{0.24}{\sqrt{N}}\right] ,\text { and } \end{aligned}$$

3 $$\begin{aligned} D^{+}= & {} \sup _{-\infty<x<+\infty }[F_{o}(x)-F_{e}(x)]\text { and } {D^{-}=\sup _{-\infty<x<+\infty }[F_{e}(x)-F_{o}(x)]}, \end{aligned}$$

where $F_{o}(x)$ is the observed cumulative distribution function (CDF) of leading digits and $F_{e}(x)$ is the CDF of the data that comply with the NBL. In addition, we calculate the ${\text {M}}$-statistic proposed by Leemis et al.^44^:

4 $$\begin{aligned} M=\max _{d=1}^{9}|o_{d}-e_{d}| \end{aligned}$$

and the *D*-statistic proposed by Cho and Gaines^47^:

5 $$\begin{aligned} D=\sqrt{\sum _{d=1}^{9}(o_{d}-e_{d})^{2}} \end{aligned}$$

where $o_{d}$ and $e_{d}$ are the proportions of observations with *d* as the first digit and proportions expected by the NBL, respectively. The latter two measures have the advantage of being less sensitive to sample size. For our main analysis, we choose to report the results for the *D*-statistic, which is the Euclidean distance from the NBL in the nine-dimensional space. All our results hold for the other three goodness-of-fit measures.

### Sample description and developmental indicators

We first collect daily data from John Hopkins University for the cumulative number of confirmed cases, the cumulative number of cured cases, and the cumulative number of deaths between January 22, 2020 and June 10, 2020. We also obtain the number of conducted tests from Our World in Data (https://ourworldindata.org/coronavirus-testing). Studies have shown that naturally occurring processes comply well with the NBL when the data grow exponentially or close to it^41, 44^. Sambridge and Jackson^29^ analyze reports from all countries affected by COVID-19 and conclude that the numbers tend to obey the NBL for infections and deaths prior to countries leveling out. Once the data reach the plateau, they are no longer expected to obey the NBL. Hence, for the data to comply with the NBL, we select the growth part using the following approach. Because data show weekly seasonality, we first compute seven-day moving averages (MA) for the new daily number of confirmed cases. Then, for each country, we identify the date with the highest MA number of new daily confirmed cases. For our main analyses, we use data before the obtained cutoff for each country. In unreported tests, we also use modified approaches. We find the maximum ratio MA(number of new daily cases)/(Days since the first case for the country) and MA(number of new daily cases)/(Days since the latest nonzero case for the country). The results are robust to alternative definitions of the cutoff date. We use moving averages only to detect the end of the growth part. For all our tests, we use actual reported data for confirmed and cured cases, death tolls, and the number of conducted tests. To determine the sample size *N* for each test, we use the corresponding number of days with non-zero data between January 22, 2020 and the cutoff date for the growth part for each country.

For developmental indicators, we select the following proxies for democratic and economic development widely used in the literature: the *Economist Intelligence Unit* Democracy Index (*EIU*) and gross domestic product per capita. Because our setup has been created during the pandemic and the testing is done on surveillance data, we use two other proxies for each country’s ability to collect and report reliable health-related data: health expenditures as a percentage of GDP, and the Universal Health Coverage Index. We download countries’ democracy indices from the *Economist Intelligence Unit* for 2019. We collect the gross domestic product (*GDP*) per capita, healthcare expenditures as percentage of GDP ($HE\_GDP$), and Universal Health Coverage Index (*UHC*) for 2017 from the World Bank (https://data.worldbank.org/). At the time we collected the data, many countries still did not have the World Bank data available for 2018 or 2019. 2017 is the latest year for which the data are available for all countries. We also acquire 2019 population data for each country from Worldometer (https://www.worldometers.info/).

From the four macroeconomic indicators (*EIU*, ln(*GDP*), $HE\_GDP$, *UHC*), we construct a single developmental index in the following way. Based on each of the four indicators, we first assign each country to a quartile rank (from 0 to 3: 0 for the lowest, 3 for the highest quartile). The developmental index for each country is the sum of quartile ranks for the four indicators. The minimum value of the index is 0, the maximum value is 12. A total of 185 countries with available data were affected by COVID-19 (see Suppl. Information).

**Figure 1:**
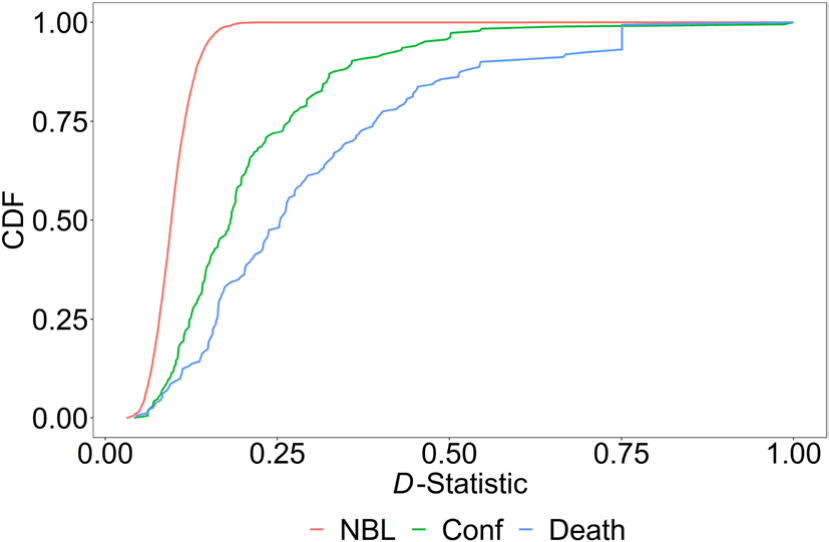
CDF plot for the *D*-statistic. The CDF plots for the *D*-statistic for the theoretical NBL, the empirical number of confirmed cases, and the empirical number of deaths based on 185 countries.

For each country, we then compute goodness-of-fit statistic using formulae (1)–(5). Using *D*-values for the 185 countries, we can plot the empirically observed distribution of the *D*-statistic and compare it to the theoretical distribution of the NBL. To plot the theoretical NBL distribution of *D*-values, we run 5,000 simulations with the sample size of 80, where first digits are randomly drawn from a discrete distribution with the NBL probabilities. Figure 1 plots the CDF of *D*-values for the theoretical NBL, the empirical number of confirmed cases, and the empirical number of deaths. We see that the empirical distribution is skewed to higher values compared to the theoretical one, and more so for the death toll. The 95th percentile for the number of confirmed cases is 0.46, and for the death toll is 0.75. Both values are much larger than either the 95th percentile value of 0.19 implied by Benford’s (1938) original data, or our simulated value of 0.15, or the reported by Goodman^34^ value of 0.25. This indicates that some countries deviate significantly from the NBL, especially when reporting deaths.

Using the conservative cutoff value of 0.25 for *D* proposed by Goodman^34^, we find that we cannot reject the NBL distribution for the entire world population for the aggregate cumulative number of confirmed cases and deaths. For individual countries, however, we find that 51 countries do not conform to the NBL when reporting the number of confirmed cases; and 86 countries do not conform to the NBL when reporting the number of deaths. In our analyses, however, we avoid country-specific conclusions based on goodness-of-fit measures that are largely dependent on a sample size and cutoff values, we aim to compare countries cross-sectionally.

Table 1 provides descriptive statistics for the major variables in our analyses. Observe that the corresponding mean goodness-of-fit measures for the number of deaths are higher than for the number of confirmed cases. This is consistent with countries, *ceteris paribus*, being more prone to misreport data on death rates.

**Table 1:** Descriptive statistics.

**Variables**	**Obs**	**Mean**	**Min**	**Med**	**Max**	**Std.**
Chi-sq. conf.	185	19.55	1.40	13.60	129.38	20.22
Kuiper conf.	185	1.48	0.39	1.37	4.70	0.73
*M* conf.	185	0.16	0.03	0.12	0.90	0.11
*D* conf.	185	0.21	0.04	0.18	0.99	0.13
Chi-sq. death	157	27.23$^{3}$	1.71	15.67	261.49	36.10
Kuiper death	157	1.67$^{3}$	0.32	1.40	5.31	1.04
*M* death	157	0.24$^{3}$	0.04	0.19	0.82	0.18
*D* death	157	0.31$^{3}$	0.07	0.26	0.90	0.11
Developmental index	155	6.05	0.00	6.00	12.00	3.66
*EIU*	164	54.83	13.20	56.45	98.70	21.91
ln(*GDP*)	178	8.68	5.68	8.66	12.03	1.44
$HE\_GDP$	174	6.44	1.18	6.23	17.06	2.57
*UHC*	175	64.44	25.00	69.00	89.00	15.68
No. of days conf.	185	61.24	1.00	61.00	136.00	30.61
No. of days death	185	33.04	0.00	23.00	124.00	28.77
ln(Population)	184	15.89	10.43	16.10	22.78	2.07

The table presents the mean values of the goodness-of-fit measures (Chi-square, Kuiper, *M*, *D*) for the cumulative number of confirmed and death cases, the developmental index and its components (*EIU*, ln(*GDP*), $HE\_GDP$, *UHC*), and other variables. The unit of observation is a country. The number of observations vary due to missing values. ^3^ indicates significance of the difference in means between confirmed and death cases at the 1% level.

**Figure 2:**
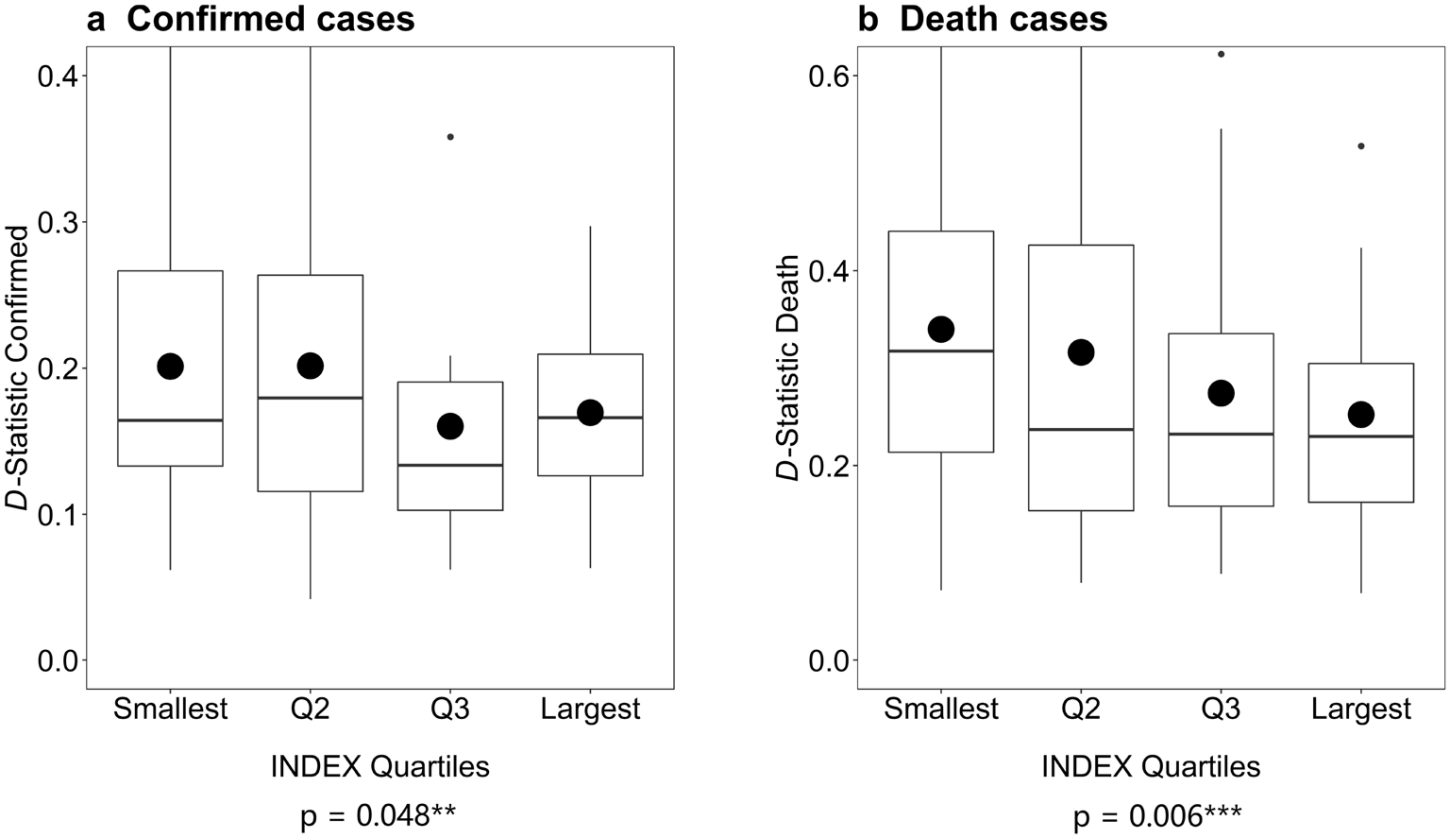
*D*-statistic and quartiles of the developmental index. The figure presents the boxplots of the *D* goodness-of-fit measure for the cumulative number of confirmed and death cases for the four quartiles of the developmental index. The unit of observation is a country. Smallest, Q2, Q3 and Largest represent the values partitioned by the 25%, 50%, and 75% quartiles of the developmental index. The boxplots represent 25%, 50%, and 75% quartiles of *D*. The dots represent the mean values. *** and ** indicate significance of the difference in means between the Smallest and the Largest quartile at the 1% and 5% level, respectively.

Figure 2 provides intuition for the main findings in our paper and plots values of the *D* goodness-of-fit measure for the four quartiles of the developmental index. The figure shows a general trend for the data to deviate less from the Newcomb-Benford distribution as we move from the smallest quartile to the largest. The trend is more pronounced for the death toll. Figure 2 also shows that the range of *D*-values for the two smallest quartiles is much wider than for the two largest quartiles. We confirm our findings with *t*-tests for the difference in means of *D*-values between the highest and the lowest quartile of the developmental index. In a univariate setting, this is consistent with our two hypotheses.

We also find that the four major economic indicators used to construct the index are highly correlated, with correlation coefficients ranging between 0.37 and 0.85 (Table 2). In unreported tests we put all economic indicators together in one equation on the right-hand-side and test for collinearity. The result shows the Condition Number is over 46, indicative of serious collinearity. The four goodness-of-fit measures are also highly correlated with each other, as are the total number of confirmed cases and the country’s population.

**Table 2:** Correlation matrix. This table presents the correlation matrix with Pearson correlation coefficients of the major variables.

Variables		(2)	(3)	(4)	(5)	(6)	(7)	(8)	(9)	(10)	(11)	(12)	(13)	(14)
Chi-sq. conf.	(1)	0.89$$^{***}$$	0.74$$^{***}$$	0.78$$^{***}$$	0.34$$^{***}$$	0.39$$^{***}$$	0.41$$^{***}$$	0.38$$^{***}$$	− 0.19$$^{**}$$	− 0.03	− 0.20$$^{**}$$	− 0.13	− 0.19$$^{**}$$	− 0.13
**Kuiper Conf.**	(2)		0.79$$^{***}$$	0.80$$^{***}$$	0.33$$^{***}$$	0.34$$^{***}$$	0.31$$^{***}$$	0.29$$^{***}$$	− 0.17$$^{*}$$	− 0.01	− 0.21$$^{**}$$	− 0.09	− 0.16$$^{*}$$	− 0.10
*M* conf.	(3)			0.95$$^{***}$$	0.27$$^{***}$$	0.29$$^{***}$$	0.48$$^{***}$$	0.47$$^{***}$$	0.00	0.09	− 0.04	0.03	− 0.01	− 0.19$$^{**}$$
*D* conf.	(4)				0.28$$^{***}$$	0.34$$^{***}$$	0.55$$^{***}$$	0.53$$^{***}$$	− 0.06	0.03	− 0.13	0.02	− 0.09	− 0.25$$^{***}$$
Chi-sq. death	(5)					0.85$$^{***}$$	0.47$$^{***}$$	0.50$$^{***}$$	− 0.33$$^{***}$$	− 0.21$$^{**}$$	− 0.28$$^{***}$$	− 0.26$$^{***}$$	− 0.31$$^{***}$$	− 0.09
Kuiper death	(6)						0.65$$^{***}$$	0.66$$^{***}$$	− 0.42$$^{***}$$	− 0.27$$^{***}$$	− 0.37$$^{***}$$	− 0.32$$^{***}$$	− 0.39$$^{***}$$	− 0.16$$^{*}$$
*M* death	(7)							0.98$$^{***}$$	− 0.17$$^{**}$$	− 0.09	− 0.21$$^{**}$$	− 0.08	− 0.19$$^{**}$$	− 0.42$$^{***}$$
*D* death	(8)								− 0.20$$^{**}$$	− 0.11	− 0.25$$^{***}$$	− 0.11	− 0.22$$^{**}$$	− 0.44$$^{***}$$
Developmental index	(9)									0.80$$^{***}$$	0.85$$^{***}$$	0.68$$^{***}$$	0.87$$^{***}$$	− 0.13
*EIU*	(10)										0.64$$^{***}$$	0.47$$^{***}$$	0.59$$^{***}$$	− 0.11
ln(*GDP*)	(11)											0.37$$^{***}$$	0.85$$^{***}$$	− 0.15$$^{*}$$
$$HE\_GDP$$	(12)												0.46$$^{***}$$	− 0.05
*UHC*	(13)													− 0.07
ln(Population)	(14)													

***, **, and * denote significance at the 1%, 5% and 10% levels, respectively.

## Results

### Goodness-of-fit and developmental index

We start with the simple ordinary least squares (OLS) regression model where our goodness-of-fit measure appears on the left-hand-side and the developmental index is on the right-hand-side:

6 $$\begin{aligned} Goodness\text {-of -}\text {fit}_{i}= & {} \beta _{0}+\beta _{1}\mathbf {Index}{}_{i}+\beta _{2}\ln (Population){}_{i}+\nonumber \\&+\beta _{3}Number\_\text {of}\_\text {Days}_{i}+\varepsilon _{i}, \end{aligned}$$

where *i* denotes a country, and Index${{_{i}}}$ is the developmental index constructed from the four economic indicators: *EIU*, ln(*GDP*), $HE{\_}GDP$, and *UHC*. We also run regressions separately for each economic indicator and find that our results are qualitatively the same. Higher values of the goodness-of-fit measures indicate greater deviation from the NBL. If more developed countries are less likely to deviate from the NBL, we expect the coefficient $\beta _{1}$ to be negative.

How well the data for each country are *expected* to obey the NBL depends on the span. For example, countries with higher populations and more confirmed cases or deaths are expected to follow the NBL more closely. To control for that, we include the natural logarithm of the country’s total population. Alternatively, we include the very correlated number of confirmed cases (or deaths). We find that the results are qualitatively and quantitatively the same when we used alternative control variables (untabulated). Even though the *D*-statistic is more independent of the sample size, goodness-of-fit measures may still be affected by the sizes of the samples used to estimate them. To control for the sample size effect, we include $Number\_\text {of}\_\text {Days}_{i}$, which is the number of days with nonzero confirmed cases (or the number of days with nonzero deaths) between January 22, 2020 and the cutoff date for the growth part for each country.

The results of estimating Eq. (6) are presented in Table 3, Panel A. Column (1) provides estimates for the cumulative number of confirmed cases. The coefficient in front of the developmental index is negative and significant. Column (2) provides estimates for the cumulative number of deaths. The coefficient is again negative and significant. The magnitude of the coefficient for the number of deaths is much higher than that for the number of confirmed cases. We find that the coefficients for the developmental index are statistically different from each other between columns (1) and (2) at the 1% significance level.

**Table 3:** First digit tests. The table presents the results of estimating Eq. 6 using OLS.

Variable	Confirmed cases	Death cases
	(1)	(2)
**Panel A. 185 countries**		
Developmental index	$$- 0.56^{***}$$ ( 0.00)	$$-1.83^{***}$$ ( 0.00)
ln(Population)	$$-1.38^{***}$$ ( 0.00)	$$-4.25^{***}$$ ( 0.00)
No. of days	$$-0.78^{***}$$ ( 0.00)	$$-2.30^{***}$$ ( 0.00)
Sample size	154	139
${Adj. R}^{2}$	13.92%	34.07%
**Panel B. 50 countries with regional data**		
Developmental index	$$-0.39^{*}$$ ( 0.07)	$$-2.38^{**}$$ ( 0.02)
ln(Population)	$-0.25$ ( 0.32)	$-2.77^{*}$ ( 0.10)
No. of days	$$-0.02^{***}$$ ( 0.00)	$$-0.13^{***}$$ ( 0.00)
Sample size	49	29
${Adj. R}^{2}$	13.77%	24.68%

The dependent variable is the *D* goodness-of-fit measure for first digits. The unit of observation is a country. Panel A shows the results for the whole dataset with 185 countries for the cumulative number of confirmed cases and death cases; panel B shows the results for 50 countries with regional data. To avoid small coefficients, we divide the development index and ln(Population) by 100 and No. of Days by 1000 for all models. Sample sizes vary due to missing values. *P*-values for a one-tailed *t*-test are in parentheses. ***, **, and * denote significance at the 1%, 5% and 10% levels, respectively.

We interpret the data as being consistent with the argument that more democratic and more developed countries are less likely to deviate from the NBL when reporting pandemic data. We also conclude that the relationship is more pronounced for the total number of deaths than for the number of confirmed cases. As predicted, the control variable $\text {ln}(Population)_{i}$ is negative in all regressions.

The results are also economically significant: an increase of one standard deviation in the developmental index, on average, results in a 0.25 increase of the standard deviation in the goodness-of-fit measure. This value is roughly the same for the number of confirmed cases and for the number of deaths. Finally, we conduct the same tests for the cumulative number of cured cases and the number of COVID-19 tests conducted. On average, we cannot reject the null hypothesis that countries’ data deviate from the NBL on cured cases or the number of conducted tests. The regression results are also not significant, indicating no systematic cross-sectional differences between countries.

Overall, we conclude that countries are most prone to misreport mortality data, slightly less so the number of confirmed cases, and that there is no evidence of systematic data falsification of cured cases or the number of tests. The cross-sectional difference between countries is also the strongest for the death toll, weaker for the total number of confirmed cases, and is insignificant for the number of cured cases and tests.

### Robustness analyses

One limitation of our analysis above is that it depends on the cutoff date for the growth part of the data. We try several different approaches. First, we use the same, “global,” cutoff value for all countries, which is 80 days since January 22, 2020 (or April 11, 2020). We pick 80 days because it corresponds to the second tercile of cutoff dates in our sample. Anything much sooner will result in too small sample sizes for many countries, especially for the ones that were affected by the pandemic later than others. Anything much later, and too many countries will have already reached their plateaus, and are not expected to obey the Newcomb-Benford law anymore. Alternatively, we try the same cutoff number of days since the first case for each country. Again, we pick the second tercile, which is 45 days since the first case for the country. The unreported results are largely consistent with our previous findings.

Another concern is that our data might be driven by countries with few cases or few data points. To test for that, we exclude countries with lower than 200 (500 and 1,000) total number of confirmed cases. We then exclude countries with fewer than 30 (40) days of nonzero cases. We also tried excluding countries with the highest 1% (5%) goodness-of-fit measures. In unreported tests we find that the results are robust in all cases. We conclude that our results are not driven by the specific pick of the cutoff date, by small countries, countries with a small number of cases, or by extreme deviations from the NBL.

### Regional data

Testing for compliance with the NBL requires sufficient data. For many countries in our analysis, the goodness-of-fit measures are calculated based on relatively small samples sizes between 40 and 140 days. Even though we control for the sample size and conduct robustness checks, making inferences from results based on such small sample sizes might be problematic. The sample size may increase significantly for a country if it reports data at a regional (state, territory, or provinces) level. Each reported value at the regional level can then be used to estimate goodness-of-fit measures, instead of using the country-level data. The method has the upside that the goodness-of-fit measures are estimated with a greater precision, though the downside is the lack of countries that collect regional data.

Fifty out of 185 countries in our sample collect regional-level data. Regional data are from the COVID-19 Coronavirus Map (https://covid19.health/). We check for data consistency between the two data sources and find the high degree of agreement. For these countries, we re-estimate the goodness-of-fit measures and re-run Eq. (6). The results are reported in Table 3, Panel B. For the cumulative number of cases, the coefficient in front of the developmental index is negative and marginally significant. For the cumulative number of deaths, the coefficient is negative and significant, even with the much smaller number of countries for this test. Again, the coefficient is statistically higher for the death toll than for the number of confirmed cases. The results are consistent with our earlier finding: more developed countries are less likely to misreport data, and the relationship is more pronounced for the number of deaths. We further conclude that our findings are not driven by the errors in the goodness-of-fit measure.

### Second digit tests

The NBL can be extended to digits beyond the first^57, 61^. Beyond the second digit, the theoretical distribution quickly converges to uniform. Diekmann^40^ notes that, when fabricating data, test subjects also naturally lean toward smaller first digits, resulting in Benford-like distributions of fabricated data. He suggests that in some cases the second-digit test may provide a clearer assessment of data manipulation. Therefore, we repeat our tests but use the second-digit goodness-of-fit measures instead of the leading digit. Our sample size drops somewhat, especially for the number of deaths, because the test requires values higher than ten. The results are presented in Table 4, again, with two panels: Panel A uses the original 185 countries, and Panel B uses the 50 countries with regional data. Column (1) provides results for the confirmed number of cases, and column (2) provides results for the number of deaths. In both panels, both columns, all coefficients in front of the developmental index are negative and significant. Again, the magnitude of the coefficients for the death toll is higher. We conclude that second-digit test results accord with our main findings.

**Table 4:** Second digit tests. The table presents the results of estimating Eq. (6) using OLS.

Variable	Confirmed cases	Death cases
	(1)	(2)
**Panel A. 185 countries**		
Developmental index	$$-0.72^{***}$$ ( 0.00)	$$-0.86^{**}$$ ( 0.02)
ln(Population)	$$-1.38^{***}$$ ( 0.01)	$$-1.99^{**}$$ ( 0.04)
No. of days	$$-2.62^{***}$$ ( 0.00)	$$-5.37^{***}$$ ( 0.00)
Sample size	151	110
${Adj. R}^{2}$	36.86%	46.99%
**Panel B. 50 countries with regional data**		
Variable	(1)	(2)
Developmental index	$$-0.35^{*}$$ (0.05)	$$-1.30^{***}$$ (0.00)
ln(Population)	0.35 (0.20)	$-1.25$ (0.11)
No. of days	$$-0.01^{*}$$ (0.08)	$$-0.10^{***}$$ (0.00)
Sample size	49	26
${Adj. R}^{2}$	2.89%	39.78%

The dependent variable is the *D* goodness-of-fit measure for second digits. The unit of observation is a country. Panel A shows the results for the whole dataset with 185 countries for the cumulative number of confirmed cases and death cases; panel B shows the results for 50 countries with regional data. To avoid small coefficients, we divide the development index and ln(Population) by 100 and No. of days by 1000 for all models. Sample sizes vary due to missing values. *P*-values for a one-tailed *t*-test are in parentheses. ***, **, and * denote significance at the 1%, 5% and 10% levels, respectively.

## Discussion

In this paper, we investigate the relationship between the accuracy of reported data and macroeconomic indicators for a set of 185 countries affected by the COVID-19 pandemic. We use the deviation from the Newcomb–Benford law of anomalous numbers as a proxy for data accuracy. For approximately one-third of countries, we document some evidence of deviations from the NBL, especially for the death toll. We find the negative relationship between the NBL goodness-of-fit measures and the index constructed from the four economic indicators. We also find that the relationship is stronger for the number of deaths than for the number of confirmed cases. Overall, we conclude that democratic regimes and more economically developed countries provide more accurate data during pandemics. We also show that the relationship holds in alternative specifications, for 50 countries that report regional data, and for second-digit tests.

Many studies in macroeconomic, accounting, finance, and forensic analysis demonstrate that human intervention and data manipulation create data sets that violate the NBL. Yet, the interpretations of our findings do not necessarily assume that deviations from the NBL are indicative of data manipulation. Several limitations to our study should be mentioned. Caution should be used when applying the NBL. Deviations from the NBL should be only treated as first filters. The aim of this paper is not to provide evidence whether a particular country manipulates data. Such claims require further investigations. We note that there is possibility that the divergence from the expected NBL distribution is not deliberate and is due to patterns of dependence between individual COVID-19 cases or structural breaks in the data. We only show that data from less developed countries is of lower accuracy and should be taken with caution.

Our paper highlights the importance of independent projects to verify data supplied by the governments. The paper also leads to a question about whether falsifying data during pandemics is a short- or long-lived strategy for governments. Further research is needed that would combine different methods that test for data manipulation, including the Newcomb-Benford law, biostatistics, moments of distributions, excess mortality rates, and social media data. Even more important is research related to methods that can prevent data manipulation and fraud during pandemics.

## Supplementary Information

Supplementary Tables.

## References

[1] Meyer, H. Experts question Russian data on Covid-19 death toll. https://www.bloomberg.com/news/articles/2020-05-13/experts-question-russian-data-on-covid-19-death-toll (2020). Accessed 16 Sep 2020.

[2] Romaniuk, S. N. & Burgers, T. Can China’s COVID-19 statistics be trusted? https://thediplomat.com/2020/03/can-chinas-covid-19-statistics-be-trusted/ (2020). Accessed 16 Sep 2020.

[3] Alwine, J. & Goodrum Sterling, F. Manipulation of pandemic numbers for politics risks lives. https://thehill.com/opinion/healthcare/499535-manipulation-of-pandemic-numbers-for-politics-risks-lives (2020). Accessed 16 Sep 2020.

[4] Economist, T. Tracking covid-19 excess deaths across countries. https://www.economist.com/graphic-detail/2020/07/15/tracking-covid-19-excess-deaths-across-countries (2020). Accessed 16 Sep 2020.

[5] Sassoon, A. M. Florida’s scientist was fired for refusing to ’manipulate’ COVID-19 data. https://www.usatoday.com/story/news/nation/2020/05/19/florida-covid-19-coronavirus-data-researcher-out-state-reopens/5218897002/ (2020). Accessed 16 Sep 2020.

[6] Speak, C. What’s the problem with Italy’s official coronavirus numbers? https://www.thelocal.it/20200403/whats-the-problem-with-italys-official-coronavirus-statistics (2020). Accessed 16 Sep 2020.

[7] Wood, G. Iran has far more coronavirus cases than it is letting on. https://www.theatlantic.com/ideas/archive/2020/03/irans-coronavirus-problem-lot-worse-it-seems/607663/ (2020). Accessed 16 Sep 2020.

[8] Cambell, C. & Gunia, A. China says it’s beating coronavirus. But can we believe its numbers? Https://time.com/5813628/china-coronavirus-statistics-wuhan/ (2020). Accessed 16 Sep 2020.

[9] Polson, D. Manipulated, agenda-driven data. https://www.redbluffdailynews.com/2020/05/04/manipulated-agenda-driven-data/ (2020). Accessed 16 Sep 2020.

[10] Aron, J. & Muellbauer, J. A pandemic primer on excess mortality statistics and their comparability across countries. https://ourworldindata.org/covid-excess-mortality (2020). Accessed 16 Sep 2020.

[11] Roukema BF (2021). Anti-clustering in the national sars-cov-2 daily infection counts. PeerJ.

[12] Goutte, S. & Damette, O. The macroeconomic determinants of COVID19 mortality rate and the role of post subprime crisis decisions. in *Available at SSRN 3610417* (2020).

[13] Dragan, A. Kak uvidet jepidemiju, esli ejo staratelno prjachut. Opyt pjati rossijskih regionov. (in Russian). https://medium.com/ (2020). Accessed 16 Sep 2020.

[14] Adsera A, Boix C, Payne M (2003). Are you being served? Political accountability and quality of government. J. Law Econ. Organ..

[15] Egorov, G., Guriev, S. & Sonin, K. Why resource-poor dictators allow freer media: A theory and evidence from panel data. *Am. Politic. Sci. Rev.* 645–668 (2009).

[16] Gehlbach S, Sonin K (2014). Government control of the media. J. Public Econ..

[17] Magee CSP, Doces JA (2015). Reconsidering regime type and growth: Lies, dictatorships, and statistics. Int. Stud. Q..

[18] Guriev S, Treisman D (2019). Informational autocrats. J. Econ. Perspect..

[19] Mitchell RB (1998). Sources of transparency: Information systems in international regimes. Int. Stud. Q..

[20] Broz, L. J. Political system transparency and monetary commitment regimes. *Int. Organ.* 861–887 (2002).

[21] Bueno de Mesquita, B., Smith, A., Siverson, R. M. & Morrow, J. D. *The Logic of Political Survival* (The MIT Press, 2003).

[22] Djankov S, McLiesh C, Nenova T, Shleifer A (2003). Who owns the media?. J. Law Econ..

[23] Fearon JD (2011). Self-enforcing democracy. Q. J. Econ..

[24] Hollyer JR, Rosendorff PB, Vreeland JR (2011). Democracy and transparancy. J. Polit..

[25] Islam R (2006). Does more transparency go along with better governance?. Econ. Polit..

[26] Lebovic JH (2006). Democracies and transparency: Country reports to the UN Register of Conventional Arms, 1992–2001. J. Peace Res..

[27] Rozenas A, Stukal D (2019). How autocrats manipulate economic news: Evidence from Russia’s state-controlled television. J. Polit..

[28] Judge G, Schechter L (2009). Detecting problems in survey data using Benford’s law. J. Hum. Resour..

[29] Sambridge M, Jackson A (2020). National COVID numbers-Benford’s law looks for errors. Nat. Corresp..

[30] Idrovo, A. J. & Manrique-Hernández, E. F. Data quality of Chinese surveillance of COVID-19: Objective analysis based on WHO’s situation reports. *Asia-Pac. J. Public Health* (2020). 10.1177/1010539520927265 PMC7231903 32408808

[31] Koch, C. & Okamura, K. Benford’s law and COVID-19 reporting. in *Available at SSRN 3586413* (2020). 10.1016/j.econlet.2020.109573 PMC7487520 32952242

[32] Peng, Y. & Nagata, M. H. Statistical analysis of the Chinese COVID-19 data with Benford’s law and clustering. https://lamfo-unb.github.io/2020/04/21/COVID-China-EN/ (2020). Accessed 16 Sep 2020.

[33] Zhang, J. Testing case number of coronavirus disease 2019 in China with Newcomb–Benford law. arXiv preprint arXiv:2002.05695 (2020).

[34] Goodman W (2016). The promises and pitfalls of Benford’s law. Significance.

[35] Nye, J. & Moul, C. The political economy of numbers: on the application of Benford’s law to international macroeconomic statistics. * BE J. Macroecon.* **7** (2007).

[36] Gonzalez-Garcia, J. *Benford’s Law and Macroeconomic Data Quality*. 2009–2010 (International Monetary Fund, 2009).

[37] Michalski T, Stoltz G (2013). Do countries falsify economic data strategically? Some evidence that they might. Rev. Econ. Stat..

[38] Newcomb S (1881). Note on the frequency of use of the different digits in natural numbers. Am. J. Math..

[39] Benford, F. The law of anomalous numbers. in *Proceedings of the American Philosophical Society*. 551–572 (1938).

[40] Diekmann A (2007). Not the first digit! Using Benford’s law to detect fraudulent scientific data. J. Appl. Stat..

[41] Formann AK (2010). The Newcomb-Benford law in its relation to some common distributions. PloS one.

[42] Hill TP (1995). A statistical derivation of the significant-digit law. Stat. Sci..

[43] Hill TP (1998). The first digit phenomenon: A century-old observation about an unexpected pattern in many numerical tables applies to the stock market, census statistics and accounting data. Am. Sci..

[44] Leemis LM, Schmeiser BW, Evans DL (2000). Survival distributions satisfying Benford’s law. Am. Stat..

[45] Morrow, J. Benford’s law, families of distributions and a test basis. in *Working Paper* (2020).

[46] Durtschi C, Hillison W, Pacini C (2004). The effective use of Benford’s law to assist in detecting fraud in accounting data. J. For. Account..

[47] Tam Cho WK, Gaines BJ (2007). Statistical fraud detection in campaign finance. Breaking the (Benford) law. Am. Stat..

[48] Horton, J., Krishnakumar, D. & Wood, A. Detecting academic fraud in accounting research: The case of Professor James Hunton. in *Available at SSRN 3164961* (2018).

[49] Cantu, F. & Saiegh, S. M. A supervised machine learning procedure to detect electoral fraud using digital analysis. in *Available at SSRN 1594406* (2010).

[50] Breunig C, Goerres A (2011). Searching for electoral irregularities in an established democracy: Applying Benford’s law tests to Bundestag elections in Unified Germany. Electoral Stud..

[51] Kaiser M (2019). Benford’s law as an indicator of survey reliability-Can we trust our data?. J. Econ. Surv..

[52] Geyer CL, Williamson PP (2004). Detecting fraud in data sets using Benford’s law. Commun. Stat.-Simul. Comput..

[53] Varian HR (1972). Benford’s law. Am. Stat..

[54] Suh, I., Headrick, T. C. & Minaburo, S. An effective and efficient analytic technique: A bootstrap regression procedure and Benford’s law. *J. For. Invest. Account.* (2011).

[55] Nigrini, M. J. *Benford’s Law: Applications for Forensic Accounting, Auditing, and Fraud Detection*. Vol. 586. (Wiley, 2012).

[56] Stambaugh C, Tipgos MA, Carpenter F, Smith M (2012). Using Benford analysis to detect fraud. Intern. Audit..

[57] Hussain, S. A. The application of Benford’s law in forensic accounting: An analysis of credit bureau data. in *Available at SSRN 1626696* (2010).

[58] Rauch B, Göttsche M, Engel S, Brähler G (2011). Fact and fiction in EU-governmental economic data. German Econ. Rev..

[59] Kalaichelvan, M. & Kai Jie Shawn, L. A critical evaluation of the significance of round numbers in major European stock indices in light of the predictions from Benford’s law. *Int. Res. J. Finance Econ.* 196–210 (2012).

[60] Rauch, B., Goettsche, M. & El Mouaaouy, F. LIBOR manipulation—Empirical analysis of financial market benchmarks using Benford’s law. in *Available at SSRN 2363895* (2013).

[61] O’Keefe, J. P. & Yom, C. Offsite detection of insider abuse and bank fraud among US failed banks 1989–2015. in *Available at SSRN 3013174* (2017).

[62] Pinilla J, López-Valcárcel BG, González-Martel C, Peiro S (2018). Pinocchio testing in the forensic analysis of waiting lists: Using public waiting list data from Finland and Spain for testing Newcomb-Benford’s law. BMJ Open.

[63] Nigrini MJ (1996). A taxpayer compliance application of Benford’s law. J. Am. Taxation Assoc..

[64] Demir, B. & Javorcik, B. K. S. *Forensics, Elasticities and Benford’s Law: Detecting Tax Fraud in International Trade* (Centre for Economic Policy Research, 2018).

[65] Marchi S, Hamilton JT (2006). Assessing the accuracy of self-reported data: An evaluation of the toxics release inventory. J. Risk Uncertain..

[66] Idrovo AJ, Fernández-Niño A, Bojórquez-Chapela I, Moreno-Montoya A (2011). Performance of public health surveillance systems during the influenza A (H1N1) pandemic in the Americas: Testing a new method based on Benford’s law. Epidemiol. Infect..

[67] Gómez-Camponovo M, Moreno J, Idrovo ÁJ, Páez M, Achkar M (2016). Monitoring the Paraguayan epidemiological dengue surveillance system (2009–2011) using Benford’s law. Biomedica.

[68] Kuiper, N. H. Tests concerning random points on a circle. *Nederl. Akad. Wetensch. Proc. Ser. A* **63**, 38–47 (1960).

[69] Stephens MA (1970). Use of the Kolmogorov-Smirnov, Cramer-Von Mises and related statistics without extensive tables. J. R. Stat. Soc. Ser. B (Methodological).

[70] Giles DE (2007). Benford’s law and naturally occurring prices in certain ebay auctions. Appl. Econ. Lett..

