# Supplementary material for: Using the Newcomb–Benford law to study the association between a country’s COVID-19 reporting accuracy and its development
Source: Sci Rep. 2021 Nov 25;11:22914. doi: 10.1038/s41598-021-02367-z (PMC8617306; doi:10.1038/s41598-021-02367-z)
Supplement: Supplementary file 1 — Supplementary Tables. [file 41598_2021_2367_MOESM1_ESM.pdf]

Using the Newcomb-Benford law to study the association between a country's COVID-19 reporting accuracy and its development

Supplementary Information

Vadim S. Balashov[1,*], **Yuxing Yan**[2], **and Xiaodi Zhu**[3]

[1]Associate Professor, Rutgers School of Business-Camden, Camden, New Jersey, 08102, United States
[2]Assistant Professor, SUNY at Geneseo, Geneseo, New York, 14454, United States
[3]Assistant Professor, New Jersey City University, Jersey City, New Jersey, 07305, United States
[*]vadim.balashov@rutgers.edu

Country	Pop.(MM)	Index	*EIU*	*GDP*	*HE_GDP*	*UHC*	Cutoff	Total Conf.	Total Death	Days Conf.	Chi Conf.	*K* Conf.	*M* Conf.	*D* Conf.	Days Death	Chi Death	*K* Death	*M* Death	*D* Death
**World**	**7815.20**	**6**	**54.40**	**18381.00**	**-**	**-**	**2020-06-08**	**7118471**	**406522**	**139**	**12.65**	**1.58**	**0.09**	**0.12**	**139**	**40.43**	**2.77**	**0.13**	**0.19**
Afghanistan	38.87	3	28.50	556.30	11.78	37.00	2020-06-05	20917	369	103	12.04	1.41	0.12	0.14	76	8.17	1.04	0.05	0.10
Albania[†]	2.88	-	58.90	4532.89	-	59.00	2020-04-25	1263	34	48	16.07	1.71	0.13	0.20	46	60.22	2.89	0.41	0.45
Algeria[†]	43.80	7	40.10	4044.30	6.37	78.00	2020-05-26	10265	715	92	4.24	0.73	0.05	0.07	76	57.57	3.06	0.19	0.29
Andorra	0.08	-	-	39134.39	10.32	-	2020-03-30	852	51	29	18.48	1.79	0.32	0.36	9	8.74	0.67	0.21	0.35
Angola	32.79	2	37.20	4095.81	2.79	40.00	2020-05-30	92	4	72	12.66	1.20	0.09	0.14	63	176.01	5.02	0.62	0.70
Antigua and Barbuda	0.10	-	-	15383.42	4.53	73.00	2020-04-07	26	3	26	38.86	2.27	0.28	0.42	0	-	-	-	-
Argentina[†]	45.17	9	70.20	14591.86	9.12	76.00	2020-06-08	23620	693	98	1.90	0.57	0.06	0.06	11	8.65	1.17	0.21	0.32
Armenia	2.96	7	55.40	3914.50	10.36	69.00	2020-06-07	13325	211	99	11.62	1.52	0.10	0.13	74	6.92	0.57	0.05	0.09
Australia[†]	25.48	12	90.90	54066.47	9.21	87.00	2020-03-30	7267	103	65	18.15	1.83	0.22	0.24	30	15.94	0.99	0.18	0.23
Austria[†]	9.00	12	82.90	47431.63	10.40	79.00	2020-03-28	16968	672	33	2.64	0.44	0.07	0.11	17	5.69	0.70	0.11	0.16
Azerbaijan	10.13	4	27.50	4147.09	6.65	65.00	2020-06-08	7876	93	100	2.73	0.46	0.03	0.04	88	5.32	0.87	0.04	0.08
Bahamas	0.39	-	-	31827.24	5.76	75.00	2020-04-26	103	11	42	18.08	1.65	0.16	0.22	1	2.32	0.98	0.70	0.75
Bahrain	1.70	7	25.50	23715.48	4.75	77.00	2020-06-06	15417	27	104	4.13	0.76	0.05	0.07	83	76.12	2.12	0.18	0.26
Bangladesh	164.59	2	58.80	1563.99	2.27	48.00	2020-06-08	68504	930	93	15.32	1.67	0.09	0.14	83	8.14	0.88	0.08	0.09
Barbados	0.29	-	-	17431.60	6.78	77.00	2020-04-07	92	7	22	12.66	1.12	0.15	0.21	3	2.67	0.81	0.21	0.31
Belarus	9.45	5	24.80	5761.75	5.93	76.00	2020-05-17	49453	276	80	11.11	1.15	0.05	0.11	48	7.99	1.25	0.12	0.17
Belgium[†]	11.59	12	76.40	44219.56	10.34	84.00	2020-04-15	59348	9606	72	30.20	2.49	0.27	0.29	36	8.23	1.03	0.13	0.16
Belize	0.40	-	-	4887.56	5.64	64.00	2020-04-13	19	2	22	3.18	0.53	0.07	0.12	0	-	-	-	-
Benin	12.10	1	50.90	1136.59	3.72	40.00	2020-05-12	339	4	58	48.45	1.67	0.18	0.29	37	41.22	3.28	0.37	0.45
Bhutan	0.77	3	53.00	3286.57	3.19	62.00	2020-06-02	59	0	89	71.54	2.25	0.14	0.26	0	-	-	-	-
Bolivia	11.66	5	48.40	3351.12	6.44	68.00	2020-06-05	13949	475	87	1.95	0.59	0.06	0.06	12	3.19	0.49	0.09	0.14
Bosnia and Herzegovina	3.28	6	48.60	5394.59	8.93	61.00	2020-04-04	2704	160	31	2.95	0.68	0.07	0.11	15	6.88	0.95	0.23	0.28
Botswana	2.35	7	78.10	7893.21	6.13	61.00	2020-05-24	42	1	56	71.53	3.17	0.41	0.45	55	127.71	5.31	0.70	0.75
Brazil[†]	212.47	10	68.60	9925.39	9.47	79.00	2020-06-08	707412	37134	104	5.32	0.94	0.04	0.07	15	13.02	1.00	0.15	0.24
Brunei	0.44	-	-	28572.15	2.37	81.00	2020-03-19	141	2	11	8.76	0.89	0.19	0.28	0	-	-	-	-
Bulgaria	6.95	7	70.30	8228.01	8.10	66.00	2020-04-27	2810	164	51	11.79	1.68	0.12	0.18	48	21.91	2.09	0.16	0.25
Burkina Faso	20.86	3	40.40	642.43	6.92	40.00	2020-04-11	890	53	33	14.09	1.78	0.16	0.23	25	14.89	1.47	0.26	0.32
Burundi	11.87	2	21.50	293.00	7.52	42.00	2020-05-18	83	1	49	39.04	1.59	0.21	0.32	36	83.59	4.33	0.70	0.75
Cabo Verde	0.56	7	77.80	3292.65	5.17	69.00	2020-06-05	567	5	78	23.74	2.17	0.12	0.21	74	29.70	2.19	0.23	0.25
Cambodia	16.70	3	35.30	1385.46	5.92	60.00	2020-03-23	126	0	57	51.46	3.24	0.42	0.46	0	-	-	-	-
Cameroon	26.50	1	28.50	1421.59	4.67	46.00	2020-06-07	8060	212	94	8.70	0.78	0.08	0.12	75	31.70	1.77	0.19	0.25
Canada[†]	37.72	12	92.20	45066.16	10.57	89.00	2020-04-22	97779	7910	88	18.05	1.13	0.09	0.13	32	24.96	1.19	0.19	0.25
Central African Republic	4.82	1	13.20	449.79	5.82	33.00	2020-06-08	1850	5	86	48.37	1.96	0.18	0.26	17	17.70	1.28	0.20	0.34
Chad	16.39	1	16.10	664.30	4.49	28.00	2020-05-09	839	70	52	11.67	0.93	0.13	0.15	12	7.37	1.01	0.16	0.26
Chile[†]	19.11	10	80.80	15037.35	8.98	70.00	2020-06-07	138846	2264	97	3.90	0.64	0.04	0.06	12	6.63	1.18	0.28	0.32
China[†]	1439.32	6	22.60	8759.04	5.15	79.00	2020-02-14	84635	4645	22	5.49	0.72	0.12	0.18	22	1.71	0.36	0.04	0.08
Colombia[†]	50.85	8	71.30	6375.93	7.23	76.00	2020-06-08	40847	1373	95	5.07	0.88	0.03	0.07	21	3.12	0.62	0.10	0.14
Comoros	0.87	3	31.50	1320.54	7.38	52.00	2020-05-24	141	2	25	24.25	1.58	0.22	0.36	19	44.12	3.19	0.70	0.75
Congo (Brazzaville)	5.51	0	31.10	1702.57	2.93	39.00	2020-05-27	683	22	74	40.53	2.44	0.16	0.23	56	51.47	2.64	0.14	0.31
Congo (Kinshasa)	89.37	0	31.10	1702.57	2.93	39.00	2020-06-04	4106	88	86	9.23	1.11	0.07	0.11	76	46.33	1.90	0.16	0.26
Costa Rica	5.09	10	81.30	11752.54	7.33	77.00	2020-06-07	1342	11	94	50.60	2.96	0.11	0.22	17	10.38	1.47	0.23	0.30
Cote d'Ivoire	-	1	40.50	1557.18	4.45	47.00	2020-06-08	3881	38	90	17.40	1.82	0.11	0.16	72	38.40	2.25	0.19	0.24
Croatia[†]	4.11	8	65.70	13412.34	6.79	71.00	2020-04-01	2247	104	37	5.48	0.77	0.12	0.14	14	17.89	1.04	0.22	0.37
Cuba	11.33	8	28.40	8541.21	11.71	83.00	2020-04-13	2200	83	33	9.19	1.25	0.12	0.18	14	26.43	1.18	0.30	0.35
Cyprus	1.21	11	75.90	26338.69	6.68	78.00	2020-04-04	970	18	27	7.84	0.66	0.08	0.16	14	9.39	0.73	0.18	0.27
Czechia[†]	10.71	10	76.90	20379.90	7.23	76.00	2020-04-02	9697	328	33	11.43	1.07	0.15	0.18	12	8.29	0.68	0.13	0.23
Denmark[†]	5.79	12	92.20	57141.06	10.11	81.00	2020-04-08	12162	593	42	4.62	0.73	0.06	0.09	26	2.99	0.40	0.05	0.09
Djibouti	0.99	1	27.70	2930.70	3.32	47.00	2020-06-02	4278	31	77	27.68	2.36	0.23	0.26	54	66.41	3.31	0.40	0.45
Dominica	0.07	-	-	7274.72	5.88	-	2020-03-26	18	0	5	5.65	0.92	0.22	0.35	0	-	-	-	-
Dominican Republic	10.84	7	65.40	7609.35	6.14	74.00	2020-06-08	20126	539	100	17.83	1.62	0.16	0.19	84	37.39	2.66	0.15	0.23

Country	Pop.(MM)	Index	*EIU*	*GDP*	*HE_GDP*	*UHC*	Cutoff	Total Conf.	Total Death	Days Conf.	Chi Conf.	*K* Conf.	*M* Conf.	*D* Conf.	Days Death	Chi Death	*K* Death	*M* Death	*D* Death
Ecuador†	17.63	10	63.30	6213.50	8.26	77.00	2020-04-24	43378	3642	55	16.66	1.51	0.12	0.19	13	8.05	0.65	0.15	0.23
Egypt	102.21	3	30.60	2440.51	5.29	68.00	2020-06-06	35444	1271	114	8.39	1.30	0.11	0.12	91	5.69	1.06	0.05	0.08
El Salvador	6.48	7	61.50	3902.24	7.23	76.00	2020-06-02	3104	56	76	6.49	1.14	0.07	0.10	8	15.67	1.08	0.25	0.41
Equatorial Guinea	1.40	2	19.20	9667.91	3.11	45.00	2020-05-19	1306	12	66	4.90	0.74	0.09	0.10	28	30.99	1.26	0.18	0.33
Eritrea	3.54	-	23.70	-	2.87	38.00	2020-04-04	39	0	15	12.23	1.05	0.23	0.32	0	-	-	-	-
Estonia†	1.33	10	79.00	20337.85	6.43	75.00	2020-04-05	1940	69	39	6.30	0.75	0.11	0.15	12	10.30	1.35	0.37	0.42
Eswatini	1.16	4	31.40	3953.09	6.93	63.00	2020-05-01	340	3	49	47.41	2.35	0.18	0.27	16	37.15	2.95	0.70	0.75
Ethiopia	114.77	0	34.40	768.43	3.50	39.00	2020-06-08	2156	27	88	10.52	1.46	0.14	0.16	65	80.47	3.35	0.26	0.39
Fiji	0.90	5	58.50	6101.03	3.50	64.00	2020-04-07	18	0	20	33.20	1.50	0.32	0.37	0	-	-	-	-
Finland†	5.54	12	92.50	46191.93	9.21	78.00	2020-04-10	7001	323	73	29.69	2.39	0.27	0.30	21	4.55	0.77	0.13	0.17
France†	65.27	12	81.20	38679.13	11.31	78.00	2020-04-16	192330	29212	84	14.46	1.38	0.09	0.15	62	14.98	1.73	0.22	0.24
Gabon	2.22	3	36.10	7212.54	2.78	49.00	2020-05-29	3101	21	77	7.04	0.96	0.09	0.11	71	63.04	3.49	0.40	0.45
Gambia	2.41	1	43.30	679.78	3.28	44.00	2020-05-08	28	1	53	49.67	2.06	0.17	0.31	47	109.13	4.92	0.70	0.75
Georgia	3.99	5	54.20	4357.01	7.60	66.00	2020-04-18	812	13	53	13.80	1.16	0.14	0.16	15	51.99	2.49	0.61	0.67
Germany†	83.77	12	86.80	44240.04	11.25	83.00	2020-04-02	186109	8695	67	20.67	1.85	0.22	0.26	25	6.72	0.72	0.10	0.16
Ghana	31.03	3	66.30	2025.89	3.26	47.00	2020-05-14	9910	48	62	6.18	0.73	0.05	0.09	55	32.70	1.51	0.12	0.22
Greece†	10.43	10	74.30	18883.46	8.04	75.00	2020-04-02	3049	182	37	20.91	1.47	0.15	0.24	23	7.84	0.99	0.09	0.16
Grenada	0.11	-	-	10163.63	4.76	72.00	2020-03-29	23	0	8	20.77	1.67	0.32	0.46	0	-	-	-	-
Guatemala	17.89	4	52.60	4470.61	5.81	55.00	2020-06-08	7502	267	87	7.50	1.01	0.06	0.09	14	12.89	1.89	0.27	0.39
Guinea	13.11	0	31.40	855.57	4.12	37.00	2020-06-03	4216	23	83	12.79	1.46	0.08	0.13	50	27.73	1.60	0.12	0.22
Guinea-Bissau	1.96	2	26.30	736.73	7.24	40.00	2020-05-10	1389	12	47	21.88	1.89	0.22	0.27	15	12.69	1.63	0.37	0.40
Guyana	0.79	6	61.50	4586.05	4.95	72.00	2020-04-17	154	12	37	14.40	1.42	0.11	0.20	0	-	-	-	-
Haiti	11.39	3	45.70	765.73	8.04	49.00	2020-06-04	3538	54	77	24.63	1.35	0.09	0.15	0	-	-	-	-
Holy See	-	-	-	-	-	-	2020-03-28	12	0	23	31.62	2.41	0.48	0.55	0	-	-	-	-
Honduras	9.89	5	54.20	2453.73	7.86	65.00	2020-05-27	6450	262	78	16.17	1.96	0.12	0.19	13	15.29	1.83	0.31	0.43
†Hungary	9.66	8	66.30	14457.61	6.88	74.00	2020-04-13	4014	548	41	3.38	0.59	0.05	0.09	30	4.34	0.87	0.13	0.16
Iceland	0.34	12	95.80	71314.77	8.33	84.00	2020-04-02	1807	10	35	6.22	0.86	0.09	0.15	17	18.44	1.76	0.35	0.40
India†	1379.20	4	69.00	1981.27	3.53	55.00	2020-06-08	265928	7473	131	44.85	2.19	0.19	0.20	90	7.06	1.27	0.09	0.11
Indonesia†	273.35	4	64.80	3836.91	2.99	57.00	2020-06-08	32033	1883	99	9.29	1.31	0.10	0.12	90	20.75	2.02	0.14	0.21
Iran†	83.93	6	23.80	5627.75	8.66	72.00	2020-04-02	173832	8351	44	4.16	0.62	0.07	0.10	44	3.68	0.78	0.10	0.11
Iraq	40.16	3	37.40	5205.29	4.17	61.00	2020-06-08	13481	370	106	6.01	0.81	0.05	0.08	97	26.40	1.99	0.08	0.15
Ireland†	4.93	10	92.40	69649.88	7.18	76.00	2020-04-16	25207	1683	48	3.85	0.50	0.05	0.09	37	7.17	1.23	0.09	0.16
Israel	9.20	11	78.60	40541.86	7.41	82.00	2020-04-03	18032	298	43	6.48	0.57	0.06	0.10	14	4.57	0.79	0.20	0.23
Italy†	60.47	12	75.20	32326.84	8.84	82.00	2020-03-26	235278	33964	56	30.64	2.21	0.21	0.29	35	2.70	0.32	0.05	0.07
Jamaica	2.96	5	69.60	5069.18	5.99	65.00	2020-04-26	599	10	47	6.08	0.49	0.06	0.10	6	12.20	1.63	0.49	0.60
Japan†	126.50	12	79.90	38331.98	10.94	83.00	2020-04-18	17060	920	88	15.46	1.25	0.13	0.17	66	18.13	1.24	0.09	0.16
Jordan	10.20	6	39.30	4162.82	8.12	76.00	2020-03-28	831	9	26	21.03	1.97	0.35	0.40	2	4.64	1.22	0.70	0.75
Kazakhstan†	18.76	4	29.40	9247.58	3.13	76.00	2020-06-03	12859	56	83	11.99	1.63	0.10	0.15	71	54.61	2.50	0.27	0.29
Kenya	53.69	3	51.80	1568.20	4.80	55.00	2020-06-08	2872	85	88	9.58	0.98	0.06	0.10	75	15.72	1.40	0.09	0.14
Kosovo	-	-	-	3948.05	-	-	2020-04-20	1263	31	26	6.44	0.81	0.08	0.15	26	26.88	2.27	0.35	0.44
Kuwait	4.27	7	39.30	29759.53	5.29	76.00	2020-05-22	32510	269	89	6.87	0.95	0.09	0.12	49	5.57	0.92	0.13	0.15
Kyrgyzstan	6.52	4	48.90	1242.77	6.19	70.00	2020-06-01	2032	23	76	13.80	1.63	0.12	0.18	60	59.26	2.63	0.25	0.37
Laos	7.27	2	21.40	2423.85	2.53	51.00	2020-03-28	19	0	5	13.60	1.06	0.33	0.50	0	-	-	-	-
Latvia†	1.89	8	74.90	15548.08	5.96	71.00	2020-04-01	1088	26	31	8.77	1.18	0.15	0.20	0	-	-	-	-
Lebanon	6.83	8	43.60	7838.34	8.20	73.00	2020-03-27	1350	30	36	10.29	1.43	0.17	0.21	18	35.69	2.48	0.32	0.51
Lesotho	2.14	5	65.40	1226.61	8.76	48.00	2020-06-03	4	0	22	27.87	2.34	0.37	0.43	0	-	-	-	-
Liberia	5.05	4	54.50	698.70	8.16	39.00	2020-06-08	370	30	85	32.09	2.30	0.12	0.21	66	46.40	2.44	0.29	0.34
Libya	6.87	-	20.20	5756.42	-	64.00	2020-06-08	332	5	77	113.06	2.84	0.30	0.33	68	70.34	2.42	0.27	0.33
Liechtenstein	0.04	-	-	-	-	-	2020-03-23	82	1	20	6.90	0.94	0.20	0.23	0	-	-	-	-
Lithuania	2.72	9	75.00	16840.94	6.46	73.00	2020-04-04	1720	71	37	5.32	0.82	0.13	0.15	15	13.90	1.28	0.14	0.26
Luxembourg	0.63	10	88.10	107361.31	5.48	83.00	2020-03-28	4040	110	29	7.35	0.82	0.15	0.19	15	30.14	1.64	0.28	0.36

Country	Pop.(MM)	Index	*EIU*	*GDP*	*HE_GDP*	*UHC*	Cutoff	Total Conf.	Total Death	Days Conf.	Chi Conf.	*K* Conf.	*M* Conf.	*D* Conf.	Days Death	Chi Death	*K* Death	*M* Death	*D* Death
Madagascar	27.64	2	56.40	515.29	5.50	28.00	2020-06-04	1094	9	77	12.67	1.62	0.15	0.19	19	32.81	1.60	0.35	0.48
Malawi	19.10	4	55.00	356.72	9.65	46.00	2020-06-03	443	4	63	17.30	1.67	0.13	0.19	58	117.60	4.16	0.43	0.51
Malaysia†	32.34	6	71.60	10254.23	3.86	73.00	2020-04-08	8329	117	75	18.67	1.53	0.16	0.18	23	12.90	1.63	0.17	0.26
Maldives	0.54	-	-	9540.63	9.03	62.00	2020-06-01	1916	8	86	38.69	2.83	0.28	0.32	34	43.76	2.62	0.29	0.36
Mali	20.21	1	49.20	828.51	3.79	38.00	2020-06-02	1547	92	70	4.96	0.87	0.06	0.08	66	28.53	1.80	0.13	0.20
Malta	0.44	12	79.50	27283.54	9.34	82.00	2020-04-11	630	9	36	11.56	1.26	0.13	0.18	4	4.51	1.02	0.32	0.39
Mauritania	4.64	1	39.20	1145.55	4.40	41.00	2020-06-07	1104	59	86	108.53	3.61	0.20	0.32	70	49.76	3.17	0.37	0.40
Mauritius	1.27	7	82.20	10484.91	5.72	63.00	2020-04-09	337	10	23	6.78	1.12	0.13	0.19	20	52.24	1.63	0.34	0.48
Mexico†	128.85	7	60.90	9278.42	5.52	76.00	2020-06-08	120102	14053	102	10.09	1.35	0.06	0.11	34	5.90	1.02	0.14	0.17
Moldova	4.03	7	57.50	3509.69	7.01	69.00	2020-06-07	9807	353	92	10.57	1.48	0.13	0.14	82	16.93	1.79	0.13	0.17
Monaco	0.04	-	-	167101.76	1.77	-	2020-03-27	99	4	28	16.56	1.50	0.20	0.27	0	-	-	-	-
Mongolia	3.27	4	65.00	3669.42	4.00	62.00	2020-05-18	194	0	70	48.69	1.84	0.18	0.32	0	-	-	-	-
Montenegro	0.63	-	56.50	7784.07	-	68.00	2020-04-06	324	9	21	9.55	1.13	0.16	0.22	15	17.72	2.14	0.29	0.43
Morocco	36.88	5	51.00	3036.33	5.25	70.00	2020-04-22	8302	208	52	7.75	0.88	0.09	0.13	44	9.04	1.36	0.18	0.20
Mozambique	31.19	2	36.50	461.42	4.94	46.00	2020-06-08	433	2	79	29.29	1.42	0.09	0.15	15	41.51	2.55	0.62	0.67
Myanmar	54.41	3	35.50	1249.83	4.66	61.00	2020-04-19	244	6	24	16.54	1.11	0.20	0.28	20	18.13	1.40	0.20	0.32
Namibia	2.54	7	64.30	5646.46	8.55	62.00	2020-03-29	31	0	16	17.04	1.20	0.24	0.36	0	-	-	-	-
Nepal	29.10	2	52.80	911.44	5.55	48.00	2020-06-08	3762	14	136	46.90	3.03	0.24	0.27	24	5.23	0.40	0.07	0.13
Netherlands†	17.13	12	90.10	48554.99	10.10	86.00	2020-04-14	47945	6035	48	5.79	1.10	0.09	0.13	40	8.48	1.13	0.12	0.16
New Zealand	5.00	12	92.60	42260.13	9.17	87.00	2020-04-05	1504	22	38	19.42	1.22	0.18	0.21	8	18.58	2.14	0.70	0.75
Nicaragua	6.62	7	35.50	2159.16	8.65	73.00	2020-05-26	1118	46	69	24.82	2.08	0.13	0.21	8	4.49	0.70	0.12	0.22
Niger	24.14	2	32.90	375.87	7.74	37.00	2020-04-11	973	65	23	5.99	0.85	0.09	0.16	18	17.75	1.63	0.37	0.44
Nigeria	205.79	2	41.20	1968.56	3.76	42.00	2020-06-04	12801	361	98	4.11	0.39	0.04	0.06	74	12.69	1.74	0.11	0.16
North Macedonia	2.08	6	59.70	5417.64	6.06	72.00	2020-06-08	3152	156	104	35.12	2.56	0.25	0.27	79	22.16	1.67	0.11	0.16
Norway†	5.42	12	98.70	75496.75	10.45	87.00	2020-03-29	8561	239	33	3.38	0.77	0.12	0.14	16	16.79	0.81	0.19	0.28
Oman	5.10	4	30.60	15130.50	3.85	69.00	2020-06-06	17486	81	104	4.57	0.84	0.05	0.08	68	7.20	0.93	0.07	0.11
Pakistan†	220.62	1	42.50	1464.99	2.90	45.00	2020-06-08	108317	2172	104	11.40	1.33	0.09	0.12	82	5.15	0.75	0.05	0.07
Palestine	5.05	-	38.90	3254.49	-	-	2020-04-07	473	3	34	6.81	1.02	0.08	0.14	13	30.19	2.68	0.70	0.75
Panama	4.31	9	70.50	15166.12	7.32	79.00	2020-06-08	16854	398	91	14.82	1.43	0.10	0.13	8	7.12	0.77	0.18	0.26
Papua New Guinea	8.94	3	60.30	2695.25	2.47	40.00	2020-04-22	8	0	34	29.77	2.22	0.20	0.33	0	-	-	-	-
Paraguay	7.13	7	62.40	5680.58	6.65	69.00	2020-05-09	1145	11	63	9.84	0.78	0.08	0.13	9	3.23	0.46	0.10	0.17
Peru†	32.94	8	66.00	6710.51	5.00	77.00	2020-06-01	199696	5571	88	6.34	1.09	0.09	0.11	11	3.38	0.40	0.08	0.15
Philippines†	109.49	4	66.40	2981.93	4.45	61.00	2020-06-04	22474	1011	127	37.35	1.90	0.17	0.19	124	35.11	2.60	0.13	0.20
Poland†	37.85	8	66.20	13856.98	6.54	75.00	2020-06-08	27160	1166	97	19.92	2.14	0.12	0.18	89	21.07	1.53	0.10	0.14
Portugal†	10.20	12	80.30	21437.35	8.97	82.00	2020-04-03	34885	1485	33	5.07	0.77	0.12	0.15	18	5.49	0.84	0.14	0.19
Qatar	2.81	4	31.90	61264.40	2.61	68.00	2020-06-03	70158	57	96	14.14	1.37	0.10	0.14	68	19.86	1.68	0.20	0.24
Romania†	19.24	7	64.90	10807.68	5.16	74.00	2020-04-17	20604	1339	52	4.42	0.83	0.07	0.11	27	7.23	0.97	0.13	0.17
Russia†	145.93	5	31.10	10750.59	5.34	74.00	2020-05-12	476043	5963	103	43.33	2.49	0.24	0.26	55	6.29	0.96	0.12	0.15
Rwanda	12.93	3	31.60	762.91	6.57	57.00	2020-04-30	451	2	48	27.79	2.24	0.28	0.33	0	-	-	-	-
Saint Kitts and Nevis	0.05	-	-	19155.43	5.04	-	2020-03-31	15	0	7	18.54	1.56	0.54	0.66	1	2.32	0.98	0.70	0.75
Saint Lucia	0.18	-	-	10039.67	4.55	68.00	2020-04-04	19	0	22	17.03	1.72	0.19	0.29	0	-	-	-	-
Saint Vincent and the Grenadines	0.11	-	-	7212.96	4.49	71.00	2020-04-09	27	0	27	31.36	2.54	0.44	0.50	0	-	-	-	-
San Marino	0.03	-	-	48494.55	7.36	-	2020-04-22	687	42	56	17.08	1.60	0.15	0.18	51	44.50	2.68	0.27	0.32
Sao Tome and Principe	0.22	-	-	1811.01	6.23	55.00	2020-05-31	513	12	56	129.38	3.39	0.40	0.50	31	29.65	1.69	0.18	0.28
Saudi Arabia†	34.78	6	19.30	20803.74	5.23	74.00	2020-05-22	105283	746	82	7.48	0.92	0.05	0.08	60	8.59	1.07	0.07	0.12
Senegal	16.71	2	58.10	1367.22	4.13	45.00	2020-05-16	4427	49	76	14.80	1.39	0.09	0.14	46	39.40	2.11	0.22	0.29
Serbia	8.74	8	64.10	6284.19	8.43	65.00	2020-04-19	11896	250	45	3.44	0.68	0.04	0.08	31	4.16	0.89	0.11	0.15
Seychelles	0.10	-	-	15683.66	5.01	71.00	2020-03-20	11	0	7	7.88	1.15	0.30	0.40	0	-	-	-	-
Sierra Leone	7.97	4	48.60	499.38	13.42	39.00	2020-05-30	1001	49	61	11.35	1.37	0.07	0.13	38	22.09	1.60	0.19	0.24
Singapore	5.85	8	60.20	60297.79	4.44	86.00	2020-04-26	38296	25	95	12.58	1.33	0.06	0.11	37	9.78	0.87	0.10	0.15
Slovakia	5.46	11	71.70	17510.09	6.74	77.00	2020-04-19	1531	28	44	6.81	0.70	0.08	0.11	14	23.83	1.85	0.47	0.53
Slovenia	2.08	12	75.00	23442.70	8.19	79.00	2020-04-02	1485	109	29	11.85	1.17	0.16	0.21	20	15.02	1.89	0.35	0.39
Somalia	15.86	-	-	309.06	-	25.00	2020-06-03	2368	84	80	14.31	1.66	0.16	0.19	57	38.97	2.50	0.16	0.26

Country	Pop.(MM)	Index	*EIU*	*GDP*	*HE_GDP*	*UHC*	Cutoff	Total Conf.	Total Death	Days Conf.	Chi Conf.	*K* Conf.	*M* Conf.	*D* Conf.	Days Death	Chi Death	*K* Death	*M* Death	*D* Death
South Africa†	59.26	9	72.40	6132.48	8.11	69.00	2020-06-08	50879	1080	96	5.88	1.03	0.04	0.08	74	11.68	0.92	0.07	0.11
South Korea†	51.27	11	80.00	29803.23	7.60	86.00	2020-03-04	11852	274	43	13.89	1.55	0.17	0.20	14	7.15	1.22	0.20	0.28
South Sudan	11.19	-	-	-	9.76	31.00	2020-05-27	1604	19	53	41.58	2.13	0.24	0.31	13	51.36	2.18	0.44	0.61
Spain†	46.75	12	82.90	28100.85	8.87	83.00	2020-03-31	241717	28752	60	16.91	1.51	0.19	0.21	29	7.61	0.69	0.09	0.14
Sri Lanka	21.41	4	62.70	4104.63	3.81	66.00	2020-05-30	1857	11	125	68.03	3.75	0.31	0.35	64	261.49	4.74	0.35	0.52
Sudan	43.78	3	27.00	3015.02	6.34	44.00	2020-05-27	6242	372	76	13.52	1.55	0.09	0.14	76	20.36	2.14	0.16	0.21
Suriname	0.59	6	69.80	5379.12	6.23	71.00	2020-06-07	128	2	86	108.04	4.70	0.49	0.54	1	4.68	1.15	0.82	0.90
Sweden†	10.10	12	93.90	53744.43	11.02	86.00	2020-06-08	45133	4694	130	26.10	2.38	0.14	0.17	90	33.28	2.49	0.13	0.20
Switzerland†	8.65	12	90.30	80450.05	12.35	83.00	2020-03-25	30972	1923	30	3.26	0.44	0.07	0.10	21	6.41	0.92	0.18	0.22
Syria	17.47	-	14.30	-	-	60.00	2020-05-28	144	6	68	70.22	2.98	0.26	0.32	61	127.47	4.68	0.42	0.54
Tajikistan	9.52	3	19.30	806.04	7.23	68.00	2020-05-21	4609	48	22	4.33	0.81	0.08	0.14	20	15.10	1.88	0.22	0.34
Tanzania	59.62	1	51.60	1004.84	3.65	43.00	2020-04-20	509	21	36	9.62	1.35	0.12	0.19	21	12.86	1.07	0.22	0.32
Thailand†	69.79	7	63.20	6578.19	3.75	80.00	2020-04-03	3119	58	73	25.04	1.92	0.15	0.20	34	41.92	2.97	0.49	0.55
Timor-Leste	1.32	4	71.90	1294.72	3.88	52.00	2020-04-20	24	0	30	33.10	2.64	0.47	0.50	0	-	-	-	-
Togo	8.27	1	33.00	626.09	6.20	43.00	2020-05-18	497	13	74	25.78	2.29	0.12	0.19	53	42.35	1.03	0.14	0.26
Trinidad and Tobago	1.40	8	71.60	16238.19	6.98	74.00	2020-03-27	117	8	14	22.08	1.82	0.30	0.43	5	17.24	1.56	0.50	0.62
Tunisia	11.81	7	67.20	3482.19	7.23	70.00	2020-04-06	1087	49	34	5.32	0.49	0.07	0.10	19	8.68	0.83	0.12	0.20
Turkey	84.28	5	40.90	10513.65	4.22	74.00	2020-04-16	171121	4711	37	7.09	0.78	0.07	0.12	31	5.64	0.79	0.11	0.16
Uganda	45.64	2	50.20	631.52	6.19	45.00	2020-06-02	646	0	74	24.86	1.49	0.14	0.18	0	-	-	-	-
Ukraine†	43.75	6	59.00	2640.68	7.00	68.00	2020-06-07	28077	805	97	17.39	1.94	0.15	0.18	87	16.01	1.60	0.12	0.15
United Arab Emirates	9.88	5	27.60	39811.63	3.33	76.00	2020-05-24	39376	281	117	21.48	1.30	0.09	0.12	66	27.21	1.94	0.23	0.25
United Kingdom†	67.87	12	85.20	40361.42	9.63	87.00	2020-04-14	288834	40680	75	41.99	1.62	0.14	0.20	40	2.92	0.50	0.07	0.08
United States of America†	330.89	12	79.60	59957.73	17.06	84.00	2020-04-10	1960897	110990	80	14.30	1.13	0.12	0.17	35	8.28	0.59	0.09	0.13
Uruguay	3.47	11	83.80	16437.24	9.30	80.00	2020-03-28	845	23	16	1.40	0.50	0.07	0.11	4	6.29	0.58	0.20	0.32
Uzbekistan	33.44	5	20.10	1826.57	6.41	73.00	2020-04-17	4440	18	34	3.41	0.75	0.07	0.11	22	35.64	2.73	0.32	0.49
Venezuela	28.44	-	28.80	-	1.18	74.00	2020-06-07	2473	22	86	17.73	1.53	0.11	0.16	7	3.59	0.78	0.12	0.20
Vietnam	97.29	4	30.80	2365.62	5.53	75.00	2020-03-28	332	0	66	27.55	2.28	0.27	0.30	0	-	-	-	-
Western Sahara	0.60	-	-	-	-	-	2020-04-05	9	1	1	9.32	1.26	0.90	0.99	0	-	-	-	-
Yemen	29.78	-	19.50	963.49	-	42.00	2020-06-05	496	112	57	15.55	1.74	0.21	0.23	37	6.02	0.70	0.11	0.14
Zambia	18.35	2	50.90	1534.87	4.47	53.00	2020-05-14	1200	10	58	12.94	1.36	0.12	0.18	43	43.03	2.05	0.27	0.33
Zimbabwe	14.85	3	31.60	1602.40	6.64	54.00	2020-06-02	287	4	75	32.17	1.94	0.20	0.23	72	213.06	4.82	0.49	0.54

Supplementary Table S.1. Original country-level data. This table shows original dataset from 185 countries. † denotes countries with regional data.

Variable	Panel A. Confirmed Cases				Panel B. Death Cases			
	Chi-squared	Kuiper	*M*	*D*	Chi-squared	Kuiper	*M*	*D*
EIU	−9.54	−0.19	−0.02	−0.05	−27.30**	−1.01***	−0.15***	−0.18***
	(0.08)	(0.22)	(0.26)	(0.09)	(0.02)	(0.00)	(0.01)	(0.00)
**ln(Population)**	−340.61***	−11.84***	−1.00**	−1.38***	−502.37***	−21.25***	−4.42***	−4.46***
	(0.00)	(0.00)	(0.02)	(0.00)	(0.01)	(0.00)	(0.00)	(0.00)
**No. of Days**	207.76***	9.31***	−0.34*	−0.66**	444.24***	14.35***	−1.34***	−0.17***
	(0.00)	(0.00)	(0.08)	(0.01)	(0.00)	(0.00)	(0.00)	(0.00)
**Sample Size**	162	162	162	162	146	146	146	146
**Adj. R^2**	10.05%	11.57%	4.67%	10.65%	13.75%	22.01%	22.18%	26.31%
**ln(*GDP*)**	−3.41***	−0.13***	−0.01**	−0.02***	−5.99***	−0.24***	−0.04***	−0.04***
	(0.00)	(0.00)	(0.01)	(0.00)	(0.00)	(0.00)	(0.00)	(0.00)
**ln(Population)**	−392.82***	−15.00***	−1.84***	−2.36***	−441.47***	−19.01***	−3.94***	−4.26***
	(0.00)	(0.00)	(0.00)	(0.00)	(0.00)	(0.00)	(0.00)	(0.00)
**No. of Days**	231.31***	9.91***	−0.34*	−0.69**	430.53***	13.99***	−1.61***	−1.99***
	(0.00)	(0.00)	(0.09)	(0.01)	(0.00)	(0.00)	(0.00)	(0.00)
**Sample Size**	176	176	176	176	152	152	152	152
**Adj. R^2**	16.06%	20.39%	17.40%	27.14%	16.50%	28.09%	27.79%	34.28%
HE_GDP	−0.53	−0.01	0.00	0.00*	−2.13**	−0.08***	−0.01**	−0.01***
	(0.17)	(0.38)	(0.19)	(0.05)	(0.03)	(0.01)	(0.01)	(0.00)
**ln(Population)**	−316.10***	−12.91***	−1.62***	−1.97***	−336.52**	−14.75***	−3.41***	−3.59***
	(0.00)	(0.00)	(0.00)	(0.00)	(0.02)	(0.00)	(0.00)	(0.00)
**No. of Days**	214.03***	9.86***	−0.42*	−0.79***	402.50***	13.75***	−1.59***	−2.01***
	(0.00)	(0.00)	(0.05)	(0.00)	(0.00)	(0.00)	(0.00)	(0.00)
**Sample Size**	173	173	173	173	148	148	148	148
**Adj. R^2**	10.94%	13.67%	16.48%	24.58%	11.90%	19.76%	22.23%	26.70%
UHC	−24.33**	−0.81**	−0.07*	−0.12**	−55.40***	−2.04***	−0.27***	−0.34***
	(0.01)	(0.01)	(0.04)	(0.00)	(0.00)	(0.00)	(0.00)	(0.00)
**ln(Population)**	−397.42***	−14.24***	−1.55***	−2.02***	−426.94***	−18.40***	−3.72***	−3.96***
	(0.00)	(0.00)	(0.00)	(0.00)	(0.01)	(0.00)	(0.00)	(0.00)
**No. of Days**	227.47***	9.82***	−0.33*	−0.66**	413.07***	13.59***	−1.50***	−1.93***
	(0.00)	(0.00)	(0.09)	(0.01)	(0.00)	(0.00)	(0.00)	(0.00)
**Sample Size**	174	174	174	174	151	151	151	151
**Adj. R^2**	14.01%	16.57%	12.78%	21.77%	16.61%	27.10%	24.50%	30.33%

Supplementary Table S.2. First digit tests. The table presents the results of estimating Equation 6 using OLS with individual indicators. The dependent variables are the four goodness-of-fit measures for first digits. The unit of observation is a country. The table uses the whole dataset of 185 countries. Panel A shows the results for the cumulative number of confirmed cases; panel B shows the results for the cumulative number of deaths. To avoid small coefficients, we divide *EIU*, *UHC*, and ln(Population) values by 100 and No. of Days values by 1,000 for all models. Sample sizes vary due to missing values. *P*-values for a one-tailed *t*-test are in parentheses. ***, **, and * denote significance at the 1%, 5% and 10% levels, respectively, after the Benjamini-Hochberg correction for multiple (=16) hypothesis testing.

Variable	Panel A. Confirmed Cases				Panel B. Death Cases			
	Chi-squared	Kuiper	*M*	*D*	Chi-squared	Kuiper	*M*	*D*
EIU	27.37	0.09	0.01	0.00	−220.31**	−4.74***	−0.27**	−0.32**
	(0.37)	(0.46)	(0.41)	(0.48)	(0.01)	(0.00)	(0.03)	(0.02)
**ln(Population)**	160.23	0.54	0.08	0.25	1316.38	22.50	−3.10	−3.02
	(0.44)	(0.49)	(0.43)	(0.32)	(0.14)	(0.13)	(0.06)	(0.07)
**No. of Days**	35.59*	0.43*	−0.02**	−0.02**	−17.72	−0.13	−0.10**	−0.12***
	(0.03)	(0.04)	(0.03)	(0.01)	(0.26)	(0.38)	(0.01)	(0.01)
**Sample Size**	50	50	50	50	30	30	30	30
**Adj. R^2**	5.09%	3.55%	7.11%	11.82%	19.78%	32.99%	24.30%	27.23%
**ln(*GDP*)**	−21.00	−0.36	−0.01	−0.01	−52.00***	−0.78**	−0.02	−0.04
	(0.09)	(0.04)	(0.23)	(0.12)	(0.00)	(0.01)	(0.20)	(0.09)
**ln(Population)**	−205.46	−4.42	−0.17	−0.37	1552.46	32.06	−2.28	−2.23
	(0.42)	(0.38)	(0.36)	(0.24)	(0.09)	(0.06)	(0.12)	(0.14)
**No. of Days**	24.22	0.26	−0.02**	−0.02**	−33.54	−0.31	−0.10**	−0.12***
	(0.10)	(0.15)	(0.02)	(0.01)	(0.10)	(0.26)	(0.02)	(0.01)
**Sample Size**	50	50	50	50	30	30	30	30
**Adj. R^2**	8.43%	9.61%	8.12%	14.38%	28.71%	25.12%	15.24%	19.81%
HE_GDP	−6.08	−0.15	0.00	0.00	−19.65***	−0.34***	−0.01	−0.02*
	(0.13)	(0.02)	(0.17)	(0.20)	(0.00)	(0.00)	(0.15)	(0.08)
**ln(Population)**	136.30	1.48	0.00	−0.07	1945.48	38.15	−1.59	−1.24
	(0.45)	(0.46)	(0.50)	(0.45)	(0.05)	(0.03)	(0.22)	(0.28)
**No. of Days**	25.84	0.22	−0.02**	−0.02**	−40.85*	−0.49	−0.10**	−0.13***
	(0.09)	(0.18)	(0.01)	(0.01)	(0.07)	(0.15)	(0.02)	(0.01)
**Sample Size**	49	49	49	49	29	29	29	29
**Adj. R^2**	7.15%	12.07%	7.43%	10.95%	31.47%	34.29%	12.41%	16.63%
UHC	−301.29	−4.29	−0.10	−0.16	−639.03***	−9.79***	−0.44*	−0.65**
	(0.03)	(0.02)	(0.07)	(0.02)	(0.00)	(0.00)	(0.06)	(0.01)
**ln(Population)**	−43.72	−1.36	−1.14	−0.32	1761.27	35.09	−2.28	−2.17
	(0.48)	(0.46)	(0.38)	(0.26)	(0.04)	(0.03)	(0.11)	(0.13)
**No. of Days**	26.07*	0.31	−0.02**	−0.02**	−21.54	−0.14	−0.09**	−0.12***
	(0.07)	(0.09)	(0.01)	(0.00)	(0.17)	(0.37)	(0.02)	(0.01)
**Sample Size**	50	50	50	50	30	30	30	30
**Adj. R^2**	11.70%	11.69%	11.37%	19.88%	45.25%	38.17%	20.96%	29.29%

Supplementary Table S.3. First digit tests for 50 countries with regional data. The table presents the results of estimating equation 6 using OLS with individual indicators. The dependent variables are the four goodness-of-fit measures for first digits. The unit of observation is a country. The table uses the dataset of 50 countries with regional data. Panel A shows the results for the cumulative number of confirmed cases; panel B shows the results for the cumulative number of deaths. To avoid small coefficients, we divide *EIU*, *UHC*, and ln(Population) values by 100 and No. of Days values by 1,000 for all models. Sample sizes vary due to missing values. *P*-values for a one-tailed *t*-test are in parentheses. ***, **, and * denote significance at the 1%, 5% and 10% levels, respectively, after the Benjamini-Hochberg correction for multiple (=16) hypothesis testing.

Variable	Panel A. Confirmed Cases				Panel B. Death Cases			
	Chi-squared	Kuiper	M	D	Chi-squared	Kuiper	M	D
EIU	−6.72	−0.30	−0.05	−0.08**	−17.49*	−0.65**	−0.07	−0.07
	(0.22)	(0.09)	(0.09)	(0.02)	(0.06)	(0.01)	(0.17)	(0.17)
**ln(Population)**	−239.27*	−6.06*	−1.14**	−1.34**	−214.75	−8.02*	−1.29	−1.77*
	(0.04)	(0.04)	(0.03)	(0.01)	(0.12)	(0.04)	(0.15)	(0.07)
**No. of Days**	−16.61	−2.69	−1.88***	−2.59***	−63.85	−5.24**	−4.05***	−5.16***
	(0.42)	(0.09)	(0.00)	(0.00)	(0.27)	(0.02)	(0.00)	(0.00)
**Sample Size**	159	159	159	159	113	113	113	113
**Adj. R^2**	1.02%	3.79%	22.94%	35.20%	1.59%	10.24%	28.08%	41.80%
**ln(*GDP*)**	−2.41*	−0.08**	−0.02**	−0.02*	−2.48**	−0.11***	−0.03**	−0.02**
	(0.03)	(0.01)	(0.01)	(0.00)	(0.02)	(0.00)	(0.01)	(0.01)
**ln(Population)**	−182.89**	−5.73**	−2.28***	−2.45***	−249.81**	−9.97**	−2.42**	−2.63**
	(0.05)	(0.02)	(0.00)	(0.00)	(0.02)	(0.00)	(0.01)	(0.01)
**No. of Days**	1.32	−2.26	−2.11***	−2.80***	−96.47	−5.97***	−4.35***	−5.46***
	(0.49)	(0.12)	(0.00)	(0.00)	(0.10)	(0.00)	(0.00)	(0.00)
**Sample Size**	170	170	170	170	116	116	116	116
**Adj. R^2**	1.74%	5.82%	30.55%	41.82%	7.48%	20.22%	35.98%	48.62%
HE_GDP	−0.21	−0.02	−0.01**	−0.01*	−2.14**	−0.07**	−0.01**	−0.02**
	(0.39)	(0.10)	(0.02)	(0.01)	(0.01)	(0.00)	(0.01)	(0.00)
**ln(Population)**	−133.83	−3.99*	−1.85***	−1.93***	−197.62	−7.95**	−1.92*	−2.15**
	(0.11)	(0.08)	(0.00)	(0.00)	(0.10)	(0.02)	(0.05)	(0.02)
**No. of Days**	−14.58	−2.90	−2.36***	−3.10***	−81.31	−5.89**	−4.62***	−5.75***
	(0.42)	(0.07)	(0.00)	(0.00)	(0.21)	(0.01)	(0.00)	(0.00)
**Sample Size**	167	167	167	167	115	115	115	115
**Adj. R^2**	−0.30%	3.97%	31.85%	43.01%	5.19%	15.88%	34.15%	47.74%
UHC	−13.32	−0.57*	−0.10*	−0.14**	−5.42	−0.57*	−0.18*	−0.17*
	(0.12)	(0.03)	(0.04)	(0.01)	(0.35)	(0.06)	(0.04)	(0.04)
**ln(Population)**	−193.19*	−5.61*	−1.94***	−2.12***	−247.38*	−9.86**	−2.61**	−2.86**
	(0.05)	(0.03)	(0.00)	(0.00)	(0.07)	(0.01)	(0.02)	(0.01)
**No. of Days**	−2.42	−2.15	−2.06***	−2.76***	−39.77	−4.51*	−4.26***	−5.34***
	(0.49)	(0.13)	(0.00)	(0.00)	(0.35)	(0.04)	(0.00)	(0.00)
**Sample Size**	169	169	169	169	117	117	117	117
**Adj. R^2**	1.01%	4.90%	28.62%	40.62%	0.38%	10.32%	33.48%	45.84%

Supplementary Table S.4. Second digit tests. The table presents the results of estimating Equation 6 using OLS with individual indicators. The dependent variables are the four goodness-of-fit measures for second digits. The unit of observation is a country. The table uses the whole dataset of 185 countries. Panel A shows the results for the cumulative number of confirmed cases; panel B shows the results for the cumulative number of deaths. To avoid small coefficients, we divide *EIU*, *UHC*, and ln(Population) values by 100 and No. of Days values by 1,000 for all models. Sample sizes vary due to missing values. P-values for a one-tailed t-test are in parentheses. ***, **, and * denote significance at the 1%, 5% and 10% levels, respectively, after the Benjamini-Hochberg correction for multiple (=16) hypothesis testing.

Variable	Panel A. Confirmed Cases				Panel B. Death Cases			
	Chi-squared	Kuiper	*M*	*D*	Chi-squared	Kuiper	*M*	*D*
	**First Digit Tests; 185 Countries**				**First Digit Tests; 185 Countries**			
**Developmental Index**	−97.45*** (0.01)	−3.21** (0.02)	−0.30* (0.06)	−0.56* (0.00)	−262.06*** (0.00)	−9.60*** (0.00)	−1.50*** (0.00)	−1.83*** (0.00)
**ln(Population)**	−326.08*** (0.00)	−11.91*** (0.00)	−1.02** (0.01)	−0.14*** (0.00)	−474.77*** (0.01)	−19.78*** (0.00)	−4.02*** (0.00)	−4.25*** (0.00)
**No. of Days**	173.86*** (0.00)	8.43*** (0.00)	−0.43** (0.05)	−0.78*** (0.00)	338.66*** (0.00)	11.11*** (0.00)	−1.82*** (0.00)	−2.30*** (0.00)
**Sample Size**	154	154	154	154	139	139	139	139
**Adj. R^2**	13.65%	13.79%	6.17%	13.92%	16.19%	27.54%	27.84%	34.07%
	**First Digit Tests; 50 Countries**				**First Digit Tests; 50 Countries**			
**Developmental Index**	−943.83* (0.05)	−18.14** (0.01)	−0.30 (0.11)	−0.39* (0.07)	−2730.79*** (0.00)	−45.99*** (0.00)	−1.66* (0.06)	−2.38** (0.02)
**ln(Population)**	−295.95 (0.39)	−7.14 (0.30)	−0.14 (0.38)	−0.25 (0.32)	222.55 (0.41)	9.31 (0.31)	−2.66 (0.11)	−2.77* (0.10)
**No. of Days**	21.07 (0.13)	0.17 (0.24)	−0.02** (0.01)	−0.02** (0.00)	−46.11** (0.02)	−0.57* (0.07)	−0.11** (0.01)	−0.13*** (0.00)
**Sample Size**	49	49	49	49	29	29	29	29
**Adj. R^2**	10.16%	15.31%	8.72%	13.77%	54.44%	53.69%	16.81%	24.68%
	**Second Digit Tests; 185 Countries**				**Second Digit Tests; 185 Countries**			
**Developmental Index**	−78.94* (0.08)	−3.16** (0.01)	−0.57** (0.01)	−0.72*** (0.00)	−78.11* (0.06)	−4.24*** (0.00)	−0.83** (0.03)	−0.86** (0.02)
**ln(Population)**	−248.99** (0.03)	−6.46** (0.03)	−1.22** (0.02)	−1.38** (0.01)	−236.29* (0.04)	−8.69* (0.01)	−1.51 (0.10)	−1.99** (0.04)
**No. of Days**	−31.64 (0.35)	−3.26* (0.06)	−1.89*** (0.00)	−2.62*** (0.00)	−92.65 (0.12)	−6.12*** (0.00)	−4.24*** (0.00)	−5.37*** (0.00)
**Sample Size**	151	151	151	151	110	110	110	110
**Adj. R^2**	2.07%	6.22%	24.22%	36.86%	4.82%	17.60%	33.05%	46.99%
	**Second Digit Tests; 50 Countries**				**Second Digit Tests; 50 Countries**			
**Developmental Index**	−1690.84*** (0.00)	−8.45* (0.06)	−0.17 (0.18)	−0.35* (0.05)	−1334.69*** (0.00)	−27.15*** (0.00)	−0.68*** (0.1)	−1.30*** (0.00)
**ln(Population)**	1014.50 (0.12)	14.19 (0.09)	0.06 (0.44)	0.35 (0.20)	−751.79 (0.14)	−22.51 (0.05)	−0.91* (0.06)	−1.25 (0.11)
**No. of Days**	233.24*** (0.00)	3.88*** (0.00)	0.00 (0.46)	−0.01* (0.08)	−24.65 (0.10)	−0.36 (0.16)	−0.06*** (0.00)	−0.10*** (0.00)
**Sample Size**	49	49	49	49	26	26	26	26
**Adj. R^2**	86.28%	90.20%	−3.18%	2.89%	41.52%	42.69%	41.49%	39.78%

Supplementary Table S.5. Developmental index and other goodness-of-fit measures. The table presents the results of estimating Equation 6 using OLS. The dependent variables are the four goodness-of-fit measures. The unit of observation is a country. Panel A shows the results for the cumulative number of confirmed cases; panel B shows the results for the cumulative number of deaths. To avoid small coefficients, we divide the developmental index and ln(Population) values by 100 and No. of Days values by 1,000 for all models. Sample sizes vary due to missing values. *P*-values for a one-tailed *t*-test are in parentheses. ***, **, and * denote significance at the 1%, 5% and 10% levels, respectively, after the Benjamini-Hochberg correction for multiple (=4) hypothesis testing.
